# Revolutionizing Veterinary Medicine: The Role of Nanoparticles in Advancing Animal Health, Nutrition and Disease Management

**DOI:** 10.1002/vms3.70528

**Published:** 2025-08-01

**Authors:** Mohsen Kazemi

**Affiliations:** ^1^ Department of Animal Science Faculty of Agriculture and Animal Science University of Torbat‐e Jam Torbat‐e Jam Iran

**Keywords:** animal nutrition, mycotoxins, nanoparticles, targeted drug delivery, veterinary medicine

## Abstract

This article presents a comprehensive evaluation of nanoparticle (NP) applications (1–100 nm) in veterinary medicine and animal science based on a rigorous assessment of studies published primarily in 2024. Key findings highlight three transformative impacts: (1) enhanced nutrition via improved mineral (zinc/selenium/iron NPs) and vitamin bioavailability, (2) advanced targeted drug delivery using liposomal/polymeric nanocarriers and (3) innovative disease control through nano‐vaccines and mycotoxin adsorbents. The evidence reveals significant improvements across species (e.g., ruminants, chickens, aquaculture and other farm animals), with documented increases in growth rates, enhanced immune responses and reduced antibiotic usage. However, critical challenges emerge regarding species‐specific dosage variations, long‐term toxicity concerns and lack of regulatory frameworks. Collectively, the evidence underscores nanotechnology's potential to revolutionize veterinary practice while emphasizing the need for robust safety protocols and tailored species guidelines.

## Graphical abstract

Nanoparticles are transforming animal care by enhancing nutrition, drug delivery and disease prevention. This study highlights their role in improving nutrient absorption in livestock, facilitating targeted drug delivery and boosting vaccine efficacy. Additionally, nanoparticles effectively reduce harmful mycotoxins in animal feeds, promoting overall health and productivity while addressing safety concerns for sustainable veterinary applications. Continued research is essential for maximizing benefits and ensuring safe usage in animal health.

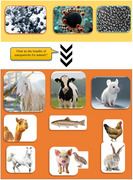

## 1 Introduction

Nanotechnology, the manipulation of matter on an atomic or molecular scale, has emerged as a transformative force across various scientific fields, including medicine, electronics and agriculture (Singh, Kumar et al. 2024). Within the realm of veterinary science and animal husbandry, the application of nanoparticles (NPs) presents innovative solutions that address key challenges in animal health, nutrition and overall productivity (Gelaye 2023). NPs, which typically range in size from 1 to 100 nm, exhibit unique physical and chemical properties that differentiate them from bulk materials (Aruna et al. 2023). NPs exhibit key characteristics such as greater surface area, higher reactivity and improved membrane penetration capacity, enabling diverse veterinary applications (Aruna et al. 2023). Their most notable nutritional application involves enhancing feed efficiency by improving nutrient bioavailability (Gelaye 2023). Conventional feeds often face absorption challenges with essential vitamins and minerals due to their bulk size, resulting in poor gastrointestinal uptake and suboptimal animal growth. NP‐based nutrient carriers have proven effective in overcoming these limitations (Altemimi et al. 2024). Mineral NPs (e.g., zinc: Zn and selenium: Se) demonstrate enhanced stability and bioavailability across species, promoting better growth and health outcomes (Malyugina et al. 2021). Additionally, NPs enable controlled nutrient release, optimizing digestive tract delivery for maximum absorption (Shafaei et al. 2017; Haydar et al. 2024). This targeted delivery is especially valuable for protecting environmentally sensitive probiotics while boosting their gut health benefits (Tolve et al. 2016; Arratia‐Quijada et al. 2024). Beyond nutrition, the innovative applications of NPs extend to veterinary diagnostics and therapeutics. Conventional drug formulations are often plagued by issues such as poor solubility and rapid clearance from the body (Adepu and Ramakrishna 2021). NPs can improve the solubility and stability of drugs, facilitating targeted delivery to specific tissues (Li et al. 2023). This targeted approach increases the therapeutic efficacy of drugs while minimizing side effects, a critical consideration in veterinary medicine. For instance, studies involving NP‐based drug delivery systems have shown promise in treating infections and chronic diseases in both human and animals, paving the way for more effective therapeutic interventions (Ranghar et al. 2013; Pramanik et al. 2021; Elumalai et al. 2024). The potential for NPs in disease prevention also stands out in veterinary medicine (El‐Sayed and Kamel 2018). The development of NP‐based vaccines has the capacity to enhance immune responses in animals (Bezbaruah et al. 2022). By using NPs as adjuvants, researchers can boost the efficacy of vaccines, leading to better disease control and prevention strategies. This aspect is particularly relevant in livestock production, where infectious diseases pose significant risks (Bezbaruah et al. 2022). Enhanced vaccination strategies utilizing NPs could contribute to higher survival rates and increased productivity in animal populations, thus supporting global food security (Bezbaruah et al. 2022). Another pressing concern in animal husbandry is the contamination of animal feed with mycotoxins and aflatoxins, toxic compounds produced by fungal contamination. Mycotoxins and aflatoxins can have detrimental effects on animal health, leading to reduced productivity and increased morbidity. Traditional methods of mycotoxin reduction often lack effectiveness and can pose additional risks to animal health (Mavrommatis et al. 2021). In contrast, NPs have demonstrated the ability to adsorb and neutralize mycotoxins, lowering their absorption in the gastrointestinal tract and mitigating their negative effects on animals (Çelik 2020). This novel application not only improves the safety of animal feed but also enhances overall animal welfare, further underscoring the potential of NPs in veterinary science. As the need for sustainable animal production practices intensifies, the role of NPs in minimizing environmental impact becomes increasingly important. By improving nutrient absorption and efficiency, NPs can reduce the overall feed requirements of livestock. This reduction has positive implications for environmental sustainability, as it decreases the resources needed for feed production. Furthermore, enhanced gut health and reduced reliance on antibiotics stemming from NP applications can lower the ecological footprint of livestock farming, aligning with the global push toward more sustainable agricultural practices. Despite the numerous advantages and potential applications of NPs in animal health and nutrition, it is crucial to address safety concerns associated with their use (Reddy et al. 2020). In this regard, a review article indicates that NPs can induce toxicity and cause significant harm to the reproductive system in animals (Brohi et al. 2017). For instance, despite their benefits, selenium NPs (SeNPs) have toxic effects, so safety considerations are necessary for effective use (Bano et al. 2022). Therefore, research into the potential toxicity of NPs to both animals and humans is essential for establishing guidelines to ensure safety in veterinary practices (More et al. 2021). Regulatory frameworks must evolve to address the unique challenges of nanomaterials in veterinary applications, including toxicity assessment, environmental risks and ethical considerations (More et al. 2021). Although nanotechnology offers transformative potential for animal health and nutrition, current guidelines remain underdeveloped and require further validation. Global harmonization of standards is essential to ensure safe and responsible implementation (Ghosh and Kumar 2024). The integration of nanotechnology into veterinary science and animal husbandry represents a forward‐thinking approach aimed at enhancing animal health, productivity and sustainability (Ali et al. 2021). Ongoing research into the design and application of NPs will play a pivotal role in overcoming the challenges associated with animal nutrition and health management. Multidisciplinary collaborations among scientists, veterinarians and industry stakeholders will be vital in translating research findings into practical applications on farms, ultimately leading to improved outcomes for both animals and producers (Reddy et al. 2023). The application of NPs in veterinary science signifies a paradigm shift in how we approach animal nutrition and health (Reddy et al. 2023). The potential for enhanced nutrient delivery, targeted therapeutics, improved vaccination strategies and mycotoxin reduction positions NPs as a powerful tool in optimizing livestock production and animal welfare (Reddy et al. 2023). As we continue to explore the applications of nanotechnology, it is imperative to maintain a focus on safety and environmental sustainability to ensure that these innovations benefit both animal populations and the broader ecosystem. Therefore, the objectives of this article are presented in Section 1.1.

### 1.1 Objectives

This narrative review comprehensively examines the multifaceted applications of NPs in veterinary medicine, with four key objectives: (1) assessing NP applications in animal nutrition and feed efficiency, particularly their role in enhancing nutrient bioavailability and digestive efficiency; (2) evaluating NP‐based drug delivery systems in veterinary therapeutics, focusing on their potential for targeted treatment and reduced side effects; (3) examining NP applications in disease prevention, including vaccine development and mycotoxin adsorption technologies; and (4) discussing critical safety concerns and regulatory considerations to ensure the responsible development and implementation of these innovative technologies. Through this comprehensive analysis, we aim to provide a balanced perspective on both the transformative potential and practical challenges of nanotechnology in animal health management.

## 2 Methodology

### 2.1 Search Strategy

A comprehensive literature search was conducted across major databases (e.g., Google Scholar, PubMed, Scopus, Elsevier, Springer, Taylor & Francis, and Wiley), covering publications from 2002 to 2025 (primarily 2024). In PubMed, the search utilized MeSH (medical subject headings) terms such as ‘NPs and veterinary medicine,’ ‘nanotechnology and animal nutrition’ and ‘drug delivery and livestock’. For other databases (Scopus, Web of Science, Elsevier, Springer, etc.), which use different indexing systems (e.g., Emtree in Embase or keyword‐based approaches), equivalent controlled vocabulary and free‐text keywords were applied to ensure consistency in retrieving relevant studies. This strategy was designed to systematically examine the applications of nanotechnology in veterinary science, encompassing NP use in animal health, nutrition and therapeutic delivery systems while maintaining methodological rigour in study selection.

### 2.2 Inclusion Criteria

The inclusion criteria for this narrative review were carefully designed to ensure the selection of high‐quality, relevant studies. This review included peer‐reviewed research articles focused on NP applications in animal health and nutrition. Studies employing laboratory, in vivo or in vitro experimental designs with clearly measurable and reported outcomes were considered. Additionally, to maintain consistency in analysis and interpretation, we restricted our review to publications available in English. These criteria were implemented to guarantee the scientific rigour and reliability of the evidence synthesized in this comprehensive evaluation of nanotechnology applications in veterinary medicine.

## 3 Nanotechnology and NPs Definition

Nanotechnology involves the design and synthesis of particles (1–100 nm) through various fabrication methods, with precise control over their size and structure (Chandrakala et al. 2022). This field, which focuses on manipulating matter at the nanoscale, has emerged as a transformative technological breakthrough with widespread scientific and industrial applications. Nanotechnology is the ability to produce new materials, tools and systems by taking control at the molecular and atomic levels and using the properties that appear on those levels (Nasrollahzadeh et al. 2019). It follows from this simple definition that nanotechnology is not a new field, but a new approach in all fields (Nasrollahzadeh et al. 2019). For nanotechnology, Malik et al. (2023) have listed applications in various fields from food, medicine, medical diagnosis and biotechnology to electronics, computing, communication, transportation, energy, environment, materials, aerospace and national security. In fact, nanotechnology is a fundamental change in the path that will lead to the manufacture of materials and tools in the future (Malik et al. 2023). The possibility of synthesizing nano building blocks with carefully controlled size and composition and then assembling them into larger structures, which have unique properties and functions, creates a revolution in materials and their production processes. The NPs cannot be recognized by the human eye and can show different physical and chemical properties than larger particles. Furthermore, they can be made of tens or hundreds of atoms or molecules with different sizes and morphologies (amorphous, crystalline, spherical, needle‐shaped, etc.) (Madkour 2019). Most of the NPs that are used commercially are in the form of dry powder, liquid or combined in an organic or aqueous solution in the form of suspension or paste (Nasiri and Janusas 2023). NPs are classified as natural or artificial based on their manufacturing method. Free (natural) NPs result from the breakdown of larger particles or controlled assembly (Khan et al. 2019). Natural phenomena (volcanic eruptions or forest fires) and many human industrial and domestic activities such as cooking, production or road and air transportation create and enter NPs into the atmosphere (Khan et al. 2019). Natural NPs also include very fine sand grains of mineral origin (such as oxides and carbonates) (Khan et al. 2019). But it should be noted that the origin of NPs is not only terrestrial and they are dumped on the earth's atmosphere by many cosmic processes (Khan et al. 2019). Semiconductor NPs can be made from any solid or liquid material, including metals, dielectrics and semiconductors. These particles may be either internally homogeneous or heterogeneous (Khan et al. 2019; Terna et al. 2021). Among the methods of artificial production of NPs, the following can be mentioned: gas compression, abrasion, chemical deposition, ion implantation, pyrolysis, radiolysis and hydrothermal synthesis (Harish et al. 2022). The properties of an NP are strongly influenced by the initial nucleation stages of the synthesis process. For example, nucleation is critical for NP size, and care must be taken to ensure that the critical radius is met in the early stages of solid formation; otherwise, the particles will revert to the liquid phase (Harish et al. 2022). The final shape of an NP is also controlled by nucleation. Possible final morphologies created by nucleation can include spherical, cubic, needle‐shaped, worm‐shaped and more (Łuczak et al. 2023). Nucleation can be controlled mainly by time and temperature and synthesis environment in general (Łuczak et al. 2023).

## 4 Nanomedicines

In the pharmaceutical industry, nanomedicine is one of the branches of nanotechnology, which can be used to make powerful and widely used tools in the field of medicine and research (Mazayen et al. 2022). In addition to making tools, the science of nanomedicine is also related to the structures of materials and drugs. This science deals with the treatment of special diseases and sensitive and professional surgeries in its sub‐branches. However, the role of nanomedicine is not to directly produce drugs but rather to optimize their delivery within the body, thereby enhancing drug efficacy (De Jong and Borm 2008). Through this approach, doctors can deliver medications precisely to the target site, avoiding inefficient distribution through conventional routes (e.g., the stomach). Nanomedicine has revolutionized targeted cancer treatment via specialized drug delivery systems. This approach involves injecting NP‐based therapeutics that selectively accumulate in tumour tissues. The mechanism works by concentrating the drugs at the cancer site, which simultaneously (1) confines malignant cells to their original location, preventing metastasis; (2) delivers focused cytotoxic effects to eradicate tumours and (3) minimizes damage to healthy surrounding tissues. Over time, this localized treatment leads to complete elimination of the cancerous cells at the target site (Seigneuric et al. 2010). Due to these advantages, nanocarrier‐based drugs have gained widespread global acceptance and hold a significant share in pharmaceutical markets.

## 5 Nanomedicine in Veterinary

**FIGURE 1:**
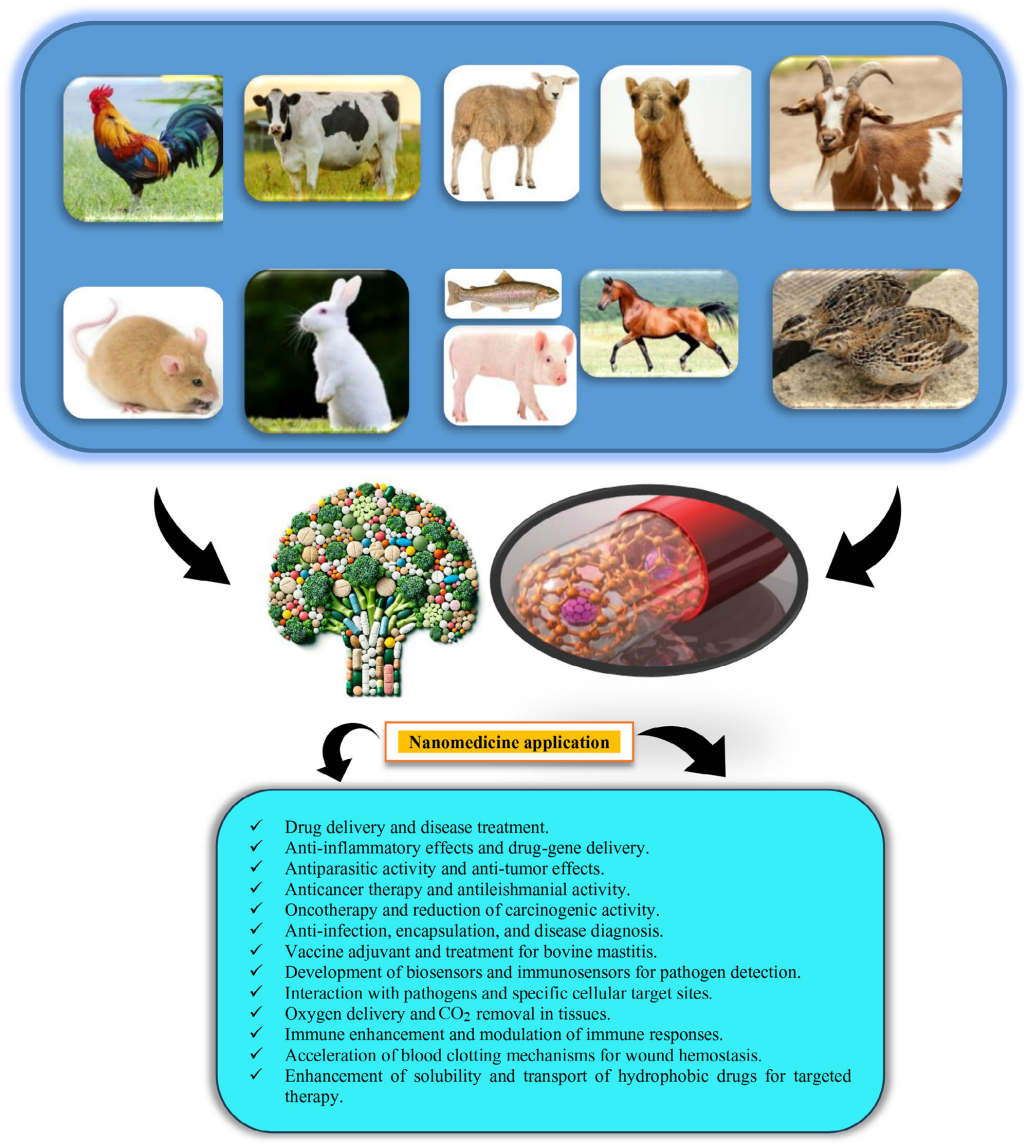
An illustration of the use of nanoparticles in animals.

Nanomedicine stands at the forefront of healthcare advancements, leveraging nanotechnology to enhance diagnosis, treatment and disease prevention. Within this rapidly growing field, NPs exhibit unique traits, such as a high surface area‐to‐volume ratio and precise targeting abilities, making them valuable in medical applications. Worldwide, the population of both pet and food‐producing animals has been steadily growing. Figure 1 illustrates the use of NPs in animals. As shown in the figure, NPs are used for drug delivery, anti‐inflammatory effects, antiparasitic/anti‐tumour activity and cancer therapy, as well as infection control, vaccine adjuvants and biosensors. Creating reliable and non‐toxic solutions that safeguard animal well‐being while ensuring ease of use, minimizing discomfort and lowering unwanted reactions remains a key concern for companion animal caregivers. Simultaneously, veterinary drug manufacturers are highly focused on maintaining the security of animal‐derived food supplies by keeping medicinal residue levels well below hazardous thresholds (Hill and Li 2017). A significant portion of pharmaceuticals currently administered in animal healthcare were originally developed for humans, often without sufficient consideration for the distinct structural, functional and metabolic variations across different species (Carvalho et al. 2020). Advancing animal health and well‐being necessitates the creation of innovative veterinary medications, which also presents a promising and profitable avenue for pharmaceutical companies. This growing focus has spurred greater investment in species‐tailored drug formulations, further driving the exploration of nanotechnology as a strategic approach for enhancing the safety and efficacy of veterinary treatments (Irache et al. 2011). Veterinary nanomedicine formulations primarily utilize two classes of nanostructured delivery systems: biologically derived and mineral‐based platforms. These include bilayer phospholipid vesicles, ultrafine dispersion systems, surfactant‐based molecular assemblies, fat‐based nanoscale carriers, macromolecular NP constructs, porous silicon dioxide nanostructures, metal‐containing nanocrystals and hyperbranched polymer networks (Oliveira et al. 2017; Sato et al. 2017; Ferreira et al. 2018; Zhang et al. 2018; Gurunathan et al. 2018; Withers et al. 2018; Sábio et al. 2019; Savić et al. 2019; Cai et al. 2019).

The primary goal of employing NP delivery systems in animal healthcare lies in their ability to target therapeutic compound distribution while decreasing required dosages, thereby mitigating severe side effects and improving tolerance during extended therapy regimens. A key advantage involves decreasing administration frequency, particularly significant in companion animal medicine where owner compliance plays a pivotal role in treatment efficacy. For livestock applications, minimizing animal restraint procedures not only alleviates stress but also enhances operational efficiency. Furthermore, nanoformulated immunizations offer the potential to extend protective immunity duration through sustained antigen release mechanisms (Moyer et al. 2016; Calderon‐Nieva et al. 2017). Nanoscale carriers have emerged as promising vehicles for anticancer therapeutics owing to their selective tumour‐targeting capabilities and capacity to minimize toxicity associated with conventional chemotherapy agents. Although cytotoxic drugs remain a mainstay treatment across various malignancies, their clinical utility is often limited by systemic toxicity and frequent development of treatment resistance. Nanotechnology provides promising solutions to these challenges. Among various delivery systems, phospholipid‐based vesicular systems stand out as particularly effective nanoplatforms. These systems enhance stabilization of hydrophobic compounds, improve tumour‐targeted biodistribution and promote selective cellular internalization. Together, these properties significantly minimize off‐target effects while maximizing therapeutic efficacy. These spherical nanostructures, comprising concentric phospholipid bilayers, typically range from 25 nm to 1 µm in diameter. Their unique architecture provides multiple benefits, including compatibility with both water‐ and lipid‐soluble pharmaceuticals, tunable surface characteristics, customizable particle dimensions and versatile functionalization potential. Phospholipid‐based nanostructures, first identified in foundational studies, have been rigorously examined in extensive scientific research. Recent advances highlight their considerable potential for various biomedical applications, especially in advancing animal health therapeutics (Sadozai and Saeidi 2013). Phospholipid‐based nanocarriers, particularly liposomes, represent the most extensively researched delivery systems in veterinary medicine. These nanostructures demonstrate superior drug delivery capabilities compared to conventional formulations. Submicron emulsions, consisting of lipid and aqueous phases stabilized by surfactants (Kumar Gupta et al. 2019; Salvia‐Trujillo et al. 2017), have proven effective for: (1) oral medication delivery via feed/water and (2) prolonged‐release injectable formulations of hydrophobic drugs (Vandamme and Anton 2010). In veterinary oncology, nanoscale systems enable targeted delivery of anticancer agents (chemotherapeutics, immunomodulators) while minimizing systemic toxicity (Lin et al. 2015). Their ability to overcome drug limitations, such as poor solubility, instability (notably in nucleic acid therapies) and narrow therapeutic indices, has opened new treatment avenues for animal cancers. Application of new nanomedicines against different diseases is shown in Table 1. As shown in the table, nanomedicine demonstrates diverse therapeutic applications across animal species: In cattle, colostrum‐derived NPs combat mastitis by modulating NF‐κB pathways, whereas silver NPs (AgNPs) effectively treat drug‐resistant *Salmonella* in sheep/goats. Poultry studies reveal chitosan‐encapsulated vaccines induce robust immunity against avian viruses (infectious bronchitis virus, H5N1). Rodent models highlight enhanced drug delivery—liposomal naringenin improves fatty liver treatment and rivastigmine formulations show superior Alzheimer's efficacy. Notably, nano‐vaccines achieve 100% recovery against foot‐and‐mouth disease in mice. These innovations showcase nanotechnology's advantages in veterinary medicine: targeted delivery, reduced side effects and enhanced treatment efficacy compared to conventional methods. Nanostructured drug carriers significantly enhance therapeutic agent transport while fundamentally altering the biodistribution and clearance patterns of anticancer medications. This transformative approach leads to markedly decreased treatment‐related adverse events and improved safety profiles (De Jong 2008).

**TABLE 1:** Application of new nanomedicine against different diseases.

Species	Nanomedicine name	Disease	Main impacts	References
Cow	Colo bovine colostrum‐derived extracellular vesicles nanomedicine	Bovine mastitis	→ The potential anti‐inflammatory effect through modulation of the NF‐κB signalling pathway	Xiong et al. (2024)
Sheep and goat	Silver nanoparticles (AgNPs)	*Salmonella*	→ The main efficacy of AgNPs in combating multidrug‐resistant *Salmonella* spp., both in vitro and in vivo, with no adverse effects	Farouk et al. (2020)
Rat	Naringenin nanoliposome	Non‐alcoholic fatty liver disease	→ The main enhancement of oral absorption of naringenin, showing comparable inhibitory effects on non‐alcoholic fatty liver disease at lower doses than crude naringenin	Chen et al. (2017)
Mice	Fat fighting oral nano‐formulation of liraglutide	Obesity	→ Improvement of drug stability under harsh gastric conditions, along with notable reductions in body weight, blood glucose and lipid levels, as well as decreased liver weight and hepatic oxidative stress	Jakhar et al. (2023)
Poultry	Inactivated infectious bronchitis virus vaccine encapsulated in chitosan NPs	Avian infectious bronchitis virus (IBV)	→ Induction of robust antibody and cell‐mediated immunity responses at mucosal sites, providing effective protection against IBV infection	Lopes et al. (2018)
Chicken	Inactivated pathogenic avian influenza‐H5N1 with selenium nanoparticles (SeNPs)	Avian influenza, H5N1 virus	→ Enhanced vaccine efficacy against pathogenic avian influenza H5N1 in chickens through SeNPs supplementation	Yehia et al. (2022)
Chicken	Formulation of Newcastle disease virus coupled calcium phosphate NPs	Newcastle	→ Potential of β‐cyclodextrin–calcium phosphate NPs as an effective vaccine delivery system for enhanced immune responses	Viswanathan et al. (2014)
Rabbit	Gelatin–epigallocatechin gallate nanoparticles with hyaluronic acid decoration (GEHNPs) as eye drops	Dry‐eye syndrome	→ Production of GEHNPS with high affinity for treating ocular inflammation and improving symptoms of dry eye syndrome through eye drops	Huang et al. (2018)
Mice	*Brucella abortus* nano‐vaccine	*Brucella abortus*	→ Emergence of new nano‐vaccines that enhance immunoglobulin production and efficacy against brucellosis	Afshari et al. (2020)
Mice	Functionalized AgNPs with Foot and mouth disease virus antigen/antibody	Foot and mouth disease (FMD)	→ Functionalized AgNPs achieve 100% recovery in infected mice from foot‐and‐mouth disease within 12 h	Sironmani (2019)
Mice and guinea pigs	Gold nanocages	FMD	→ Significant enhancement of the immune response against the FMD virus using virus‐like particles combined with gold nanocages	Teng et al. (2021)
Rat	Rivastigmine liposomes (RLs)	Alzheimer	→ Superior efficacy of RLs compared to rivastigmine solution in improving spatial memory and normalizing biochemical parameters in an Alzheimer's disease model	Ismail et al. (2013)

Abbreviations: NF‐Κb, nuclear factor Kappa‐light‐chain‐enhancer of activated B cells; NPs, nanoparticles.

**TABLE 2:** Effect of nanoparticles (NPs) on reducing pollution caused by mycotoxins (M) and aflatoxins (A).

Items tested	Nanoparticle name	M/A name	Main effects	References
Maize crops	NPs extracts of *Agaricus* spp.	*Aspergillus flavus* aflatoxin B1	→ Effectiveness of NPs in protecting maize crops from infection and aflatoxin B1 production by *A. flavus*	Hussein and Owied (2024)
Contaminated animal feed	Fungal‐produced selenium nanoparticles (SeNPs)	*Fusarium oxysporum* (CCASU‐2023‐F9)	→ Fungal‐produced SeNPs effectively inhibited pathogens and mycotoxins in animal feed, enhancing feed quality	Gharieb et al. (2024)
Beef sausage	Pumpkin seed oil NPs	*A. flavus*	→ Pumpkin seed oil and its NPs effectively inhibited *A. flavus* growth and reduced aflatoxin levels in beef sausage, offering a natural preservative solution for food safety	Khalid and Elokle (2025)
Beef burger patties	Cinnamon essential oil nanoparticles (CEO‐NPs)	*A. flavus*	→ CEO‐NPs completely inhibited *A. flavus* growth and reduced aflatoxins by 68.2% in beef burgers, demonstrating strong potential as a natural food preservative	Bakr et al. (2024)
Peach fruit	Iron oxide nanoparticles (Fe_2_O_3_NPs)	Aflatoxins B1 and B2	→ Significant reduction of aflatoxin production in Fe_2_O_3_ NP‐treated peach fruit	Akbar et al. (2024)
Milk	Magnetized chitosan nanoparticles composed of molybdenum disulphide (Fe_3_O_4_/Cs/MoS2/LacNPs)	Aflatoxin M1	→ Highest removal percentage of aflatoxin M1 (68.5%) achieved by Fe_3_O_4_/Cs/MoS2/LacNPs	Rezagholizade‐shirvan et al. (2024)
Vegetable oil and peanut milk	Magnetic reduced graphene oxide composite (Fe_3_O_4_@rGO)	Aflatoxin B1	→ Fe_3_O_4_@rGO effectively adsorbed aflatoxin B1 (82.64 mg/g) in food while preserving nutritional quality, demonstrating strong potential for food safety applications	Zhang, Zhou et al. (2024)
Ras cheese	Nisin NPs	*A. flavus*	→ Strong antifungal activity of nisin NPs (0.0625 mg/mL) against *A. flavus*, more effective than pure nisin (0.5 mg/mL)	Abd‐Elhamed et al. (2024)
Peanut	Nano‐encapsulated *Cuminum cyminum* essential oil (Ne‐CEO)	Mould species and aflatoxin B1	→ Ne‐CEO exhibited superior antimicrobial effects compared to non‐encapsulated *Cuminum cyminum* essential oil, inhibiting growth of selected mould species and aflatoxin B1 secretion	Kumar, Raghuvanshi et al. (2024)
Corn kernels	Silver nanoparticles (AgNPs)	*A. flavus*	→ AgNPs repressed the mycelial growth and germination of *A. flavus* spores more than the crude extract, AgNO_3_ and the fungicide	Elazab et al. (2024)
Maize seed	Green‐synthesised copper oxide–zinc oxide hybrid NPs (Green ZnO–CuONPs)	*A. flavus*	→ Green ZnO–CuONPs effectively inhibited *A. flavus* and enhanced maize seed germination under AFB1 contamination	Ngwenya et al. (2025)
Soil samples	AgNPs	Mycotoxigenic fungi *A. flavus* ATCC 11498 and *Aspergillus ochraceus* ATCC 60532	→ Biogenic AgNPs completely inhibited the production of total aflatoxins and ochratoxin A at concentrations less than 8 µg/mL	Abd El‐Ghany et al. (2023)
NIH3T3 cells and haemolysis test	Mesoporous silica nanoparticles (MSNPs)	Aflatoxin B1	→ MSNPs can reduce cell toxicity produced by aflatoxin B1 due to its potential to adsorb	Savi et al. (2022)
Culture medium	Titanium dioxide nanoparticles (TiO_2_–NPs)	Aflatoxin B1	→ TiO_2_–NPs in combination with selected yeast strains have a high ability to remove aflatoxin B1 from the medium	Nasiri Poroj et al. (2023)
In vitro (wheat seeds) and in vivo (Albino rats)	*Juglans‐regia*‐Mediated AgNPs	*Aspergillus ochraceus*	→ The production of aflatoxins was successfully inhibited by AgNPs	Naqvi et al. (2023)
Mice	SeNPs	Aflatoxin B1	→ SeNPs could inhibit aflatoxin B1‐induced damage to the testis and improve sperm parameters	Asadpour et al. (2020)
Pistachio seed	AgNPs and quinoa peptide enriched nanocomposite films	Aflatoxin B1 and total aflatoxins (B1, B2, G1, and G2)	→ The combined use of AgNPs and quinoa peptides in pistachio packaging reduced aflatoxins and increased the shelf life of raw pistachio	Mahdavi‐Yekta et al. (2022)

## 6 NPs, Mycotoxins and Aflatoxins

Mycotoxins refer to all toxins produced by fungi. These toxins can have negative effects on the health of humans and animals and encompass various groups, including zearalenone, ochratoxin and others (Awuchi et al. 2021). In contrast, aflatoxins are a specific type of mycotoxin mainly produced by fungi of the genus *Aspergillus*, particularly *Aspergillus flavus* and *Aspergillus parasiticus* (Benkerroum 2020). Mycotoxins are widely produced in fruits, grains, nuts and various agricultural products; however, aflatoxins are frequently found in food items such as almonds, hazelnuts, corn and oilseeds (such as sesame) (Awuchi et al. 2021). The effects of NPs on reducing pollution caused by mycotoxins and aflatoxins are exhibited in Table 2. The table demonstrates NPs effectively reduce mycotoxin and aflatoxin contamination across various applications. In agriculture, NPs like Agaricus extracts prevent aflatoxin production in maize, whereas SeNPs inhibit mycotoxins in animal feed. For food products, pumpkin seed oil and cinnamon NPs significantly reduce aflatoxin levels in meat products by 32%–68%. Magnetic chitosan NPs (CNPs) remove nearly 70% of aflatoxin M1 from milk, and AgNPs completely inhibit toxin production in soil at concentrations below 8 mg/mL. These nano‐based solutions show superior performance compared to conventional methods, providing effective protection throughout the food production chain from crops to final products. Mycotoxins can cause a range of adverse effects, including toxicity and chronic diseases. Aflatoxins, in particular, are more dangerous due to their high carcinogenic activity and liver toxicity, being directly associated with liver diseases and liver cancer (Benkerroum 2020). Mycotoxins consist of a broad spectrum of chemical compounds, whereas aflatoxins are specific and defined compounds with a particular chemical structure (El‐Sayed et al. 2022). Therefore, although aflatoxins are a type of mycotoxin, not all mycotoxins are aflatoxins. Recently, NPs have emerged as effective tools for the detection of aflatoxins and mycotoxins in various agricultural products. For example, the importance of NP‐modified aptasensors‐sensors that utilize aptamers for their sensitivity and selectivity in mycotoxin detection has been highlighted (Bahari et al. 2024). Furthermore, the development of reliable and sensitive detection and control methods for mycotoxins, utilizing novel nanomaterials, is crucial for ensuring food safety and public health due to the harmful effects of mycotoxin contamination (Lin et al. 2022). Additionally, the development of magnetic NP‐assisted biosensors for mycotoxin detection in food crops, which is critical for addressing the global challenge of mycotoxin contamination and enhancing sensitivity and accuracy in monitoring their presence, has been reviewed in a study (Gao et al. 2023). This study demonstrated that magnetic NP‐based biosensors show significant potential for mycotoxin detection in the food chain, owing to their enhanced sensitivity and accuracy (Gao et al. 2023). The key point of a study was that nanotechnology, particularly through the use of carbon‐based nanomaterials and polymeric NPs, offers promising and innovative strategies for effectively eliminating mycotoxins from agricultural products to mitigate their harmful health effects on humans and animals (Horky et al. 2018). In other study, it was found that zinc oxide NPs (ZnONPs) bioproduced by the *bacterium Pseudomonas poae* effectively inhibit the growth of the fungus *Fusarium graminearum* and significantly reduce mycotoxin production, including the suppression of deoxynivalenol (DON) synthesis and downregulation of the FgTRI gene (Ibrahim et al. 2024). The study demonstrated that ZnONPs protect wheat plants from disease by causing damage to the fungal cell wall and generating reactive oxygen species (ROS), as well as preventing the fungus from colonizing the wheat plants (Ibrahim et al. 2024). In another study, the antifungal and antimycotoxigenic activity of fullerenol NPs (FNPs) was investigated against the growth of *A. flavus*, isolated from a real food sample, and the production of aflatoxins B1 and B2 (Živančev et al. 2024). The findings revealed that FNPs inhibited *A. flavus mycelium* growth by up to 36% after 14 days at optimal concentrations and reduced aflatoxins B1 levels by up to 60% in artificially inoculated corn flour after the same period (Živančev et al. 2024). Strong correlations were observed between aflatoxin concentrations and *A. flavus mycelium* mass, indicating the potential of FNPs as effective agents in managing fungal growth and mycotoxin production (Živančev et al. 2024). Recurrent contamination of feed materials with mycotoxigenic fungi poses risks to both farm animals and humans. Bio‐synthesized nanomaterials are seen as promising solutions to combat fungal threats in feed and food, particularly when derived from sustainable sources (Tayel et al. 2024). Recently, nanotechnologies have significant potential to enhance the efficiency of treatment and diagnosis of diseases. Metal NPs such as iron, Zn, silver (Ag) and Se exhibit notable antimicrobial activities that inhibit the growth of mycotoxigenic moulds and prevent the production of their mycotoxins. The antifungal effects of these metal NPs result from damaging the cell walls of microbial cells, leading to cell death (Hassan et al. 2020). Additionally, zinc NPs (ZnNPs) and SeNPs have been used to mitigate the toxic effects of aflatoxins in rabbits and rats, showing hepatoprotective effects by scavenging free radicals and increasing antioxidant activity in the liver against mycotoxin adverse effects. Moreover, low doses of metal NPs were found to be more effective than low or high doses of bulk metal materials in reducing the carcinogenic effects of mycotoxins in animals (Hassan et al. 2020). Recently, synergistic and combination therapies involving metal NPs, chemical antibiotics, herbs and ozone fumigation have been successfully employed for treating animal diseases. These therapies reduce NP doses, helping to avoid toxic levels of nanomaterials in animal feed. Additionally, synergistic treatments help overcome microbial resistance to traditional antibiotics, revealing enhanced antimicrobial and antimycotoxin activities of NPs for treating human and animal diseases (Hassan et al. 2020). Hence, a study focused on the phycosynthesis of SeNPs using *Cystoseira myrica* algal extract (CE) and their combination with CNPs to create potential antifungal nanocomposites aimed at controlling *A. flavus* isolates in fish feed (Tayel et al. 2024). The study results indicated that the CNPs/CE/SeNPs nanocomposite serves as an effective and biosafe antifungal agent for controlling *A. flavus* strains and preventing fungal growth in fish feed (Tayel et al. 2024). AgNPs are effective nano‐fungicides against *Ustilaginoidea virens*, the cause of rice false smut disease, by inhibiting fungal growth and altering gene expression related to mycelial growth and toxin production (Wen et al. 2023). The cell‐free culture filtrate of *Fusarium chlamydosporum* and *Penicillium chrysogenum* successfully synthesized AgNPs, which demonstrated significant antifungal activity and effectively inhibited mycotoxin production, with low minimum inhibitory concentrations against *Aspergillus* species (Khalil, Abd El‐Ghany et al. 2019). A key finding is that these AgNPs showed promising potential as antifungal agents while maintaining relatively low cytotoxicity to human cells (Khalil, Abd El‐Ghany et al. 2019). In addition, in another study, AgNPs were evaluated as a new antifungal agent for controlling toxigenic *Fusarium* species and reducing mycotoxin contamination in cereal grains (Tarazona et al. 2019). The results indicated that AgNPs could effectively inhibit the growth and mycotoxin production of these fungi, enhancing their potential use in the agricultural and food industries (Tarazona et al. 2019). Furthermore, a study investigated the antifungal activity of AgNPs against both fungicide‐sensitive and resistant strains of *F. graminearum*, revealing that AgNPs effectively inhibit fungal growth and influence mycotoxin production, particularly DON (Jian et al. 2022). A key finding is that combining AgNPs with DON‐reducing fungicides could enhance management strategies for Fusarium head blight in cereal crops (Jian et al. 2022). Aflatoxin B1, a toxin derived from plants, can cause oxidative stress in fish. Se, a micromineral known for its antioxidant properties, was evaluated for its protective effects against aflatoxin B1‐induced oxidative stress (Sherif and Zommara 2023). In this study, Nile tilapia weighing approximately 32.2 ± 1.7 g was randomly assigned to six groups for an 8‐week feeding trial, receiving SeNPs at a concentration of 1 mg/kg in their feed alongside aflatoxin B1 at 500 µg/kg. It was found that SeNPs can effectively mitigate oxidative stress and restore immune function in Nile tilapia exposed to aflatoxin B1 (Sherif and Zommara 2023). A key takeaway is that SeNPs not only alleviate the harmful effects of aflatoxin B1 but also enhance the fish's resistance to infections, indicating their potential as a protective supplement in aquaculture (Sherif and Zommara 2023). Aflatoxin M1 and ochratoxin A (OTA) are dangerous mycotoxin byproducts that contaminate food, presenting significant health hazards to both humans and animals (Abdelnaby et al. 2021). In this context, the study established a reliable HPLC method for detecting aflatoxin M1 and OTA in contaminated milk and evaluated the detoxification efficacy of bentonite, date pit and CNPs (Abdelnaby et al. 2021). It was found that bentonite and date pit were significantly more effective in removing aflatoxin M1 (68% and 56%, respectively) and OTA (64% and 52%) compared to CNPs, with minimal impact on the nutritional components of milk. The key takeaway is that bentonite and date pit are effective adsorbents for detoxifying aflatoxin M1 and OTA from contaminated milk (Abdelnaby et al. 2021). Silymarin, derived from the milk thistle plant (*Silybum marianum*), is one of the most well‐known and extensively studied plant‐based feed additives, rich in flavonolignans and the flavonoid taxifolin (Federico et al. 2017). It enhances the metabolic activity of liver cells and promotes ribosomal RNA synthesis, leading to increased protein production (Vargas‐Mendoza et al. 2021). Research has demonstrated that silymarin can restore both endogenous antioxidant enzymes and non‐enzymatic antioxidants, such as vitamins E and C, in the livers of stressed laying hens (Pradeep et al. 2007) and reduce lipid and protein oxidation in broiler chickens (Alhidary et al. 2017). Due to its low water solubility, silymarin is often formulated into NPs to improve its bioavailability and absorption in the intestinal tract. In this regard, a study examined the impact of silymarin‐nanohydrogle on the growth performance and serum biochemical parameters of Japanese quail chicks fed a diet contaminated with aflatoxins (Khaleghipour et al. 2020). The results indicated that the inclusion of 500 mg/kg silymarin‐nanohydrogle in drinking water significantly improved daily weight gain (DWG) and feed intake, while also mitigating the adverse effects of aflatoxins on liver function and overall health (Khaleghipour et al. 2020). The key finding is that silymarin‐nanohydrogle can effectively counteract the negative impacts of aflatoxin contamination in quail diets (Khaleghipour et al. 2020). The results of a study demonstrated that magnetic nanostructured agents can effectively remove up to 90% of mycotoxins and cyanotoxins from contaminated food and feed, highlighting the significant potential of nanotechnology in mitigating natural toxin contamination (González‐Jartín et al. 2024). The key finding is that smaller composites show enhanced adsorption capacities, particularly in complex matrices (González‐Jartín et al. 2024). Ochratoxins A, B and C are mycotoxins produced by filamentous fungi, particularly *Aspergillus* and *Penicillium* species, under moderate environmental conditions (Jeswal and Kumar 2015). Among these, OTA is the most significant, known for its nephrotoxic, hepatotoxic, cytotoxic and immune‐suppressing effects, as well as its ability to reduce poultry productivity (Murugesan et al. 2015). Additionally, OTA poses serious health risks to humans, including teratogenic, carcinogenic and mutagenic effects, along with endocrine‐disrupting impacts across all animal species (Al Shap et al. 2022). In this context, a study found that metal nanocomposite effectively mitigates the adverse effects of OTA in broilers, leading to significant improvements in body weight gain (BWG), serum parameters and kidney function (Al Shap et al. 2022). The key takeaway is that both low and high doses of metal nanocomposite can counteract OTA toxicity without introducing significant metal residues in the liver and muscle (Al Shap et al. 2022).

## 7 Impacts of NPs in Different Animals

### 7.1 Impacts of NPs in Ruminants

**FIGURE 2:**
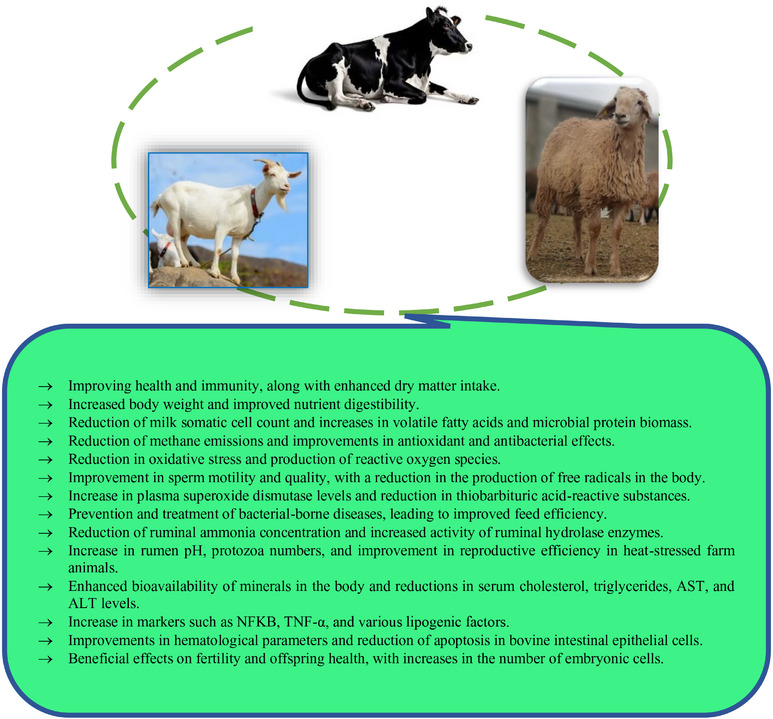
Main effects of nanoparticles in ruminant animals. ALT, alanine aminotransferase; AST, aspartate aminotransferase; NFKB, nuclear factor kappa B; TNF‐α, tumour necrosis factor alpha.

NPs can be mainly categorized into organic and inorganic materials, based on their chemical characteristics. In the livestock sector, the nutritional values of feed can be enhanced by using organic NP (such as proteins, fats and sugar molecules) supplementation (Ezzat Abd et al. 2017). The main effects of NPs in ruminant animals are shown in Figure 2. As shown in this figure, NPs improve ruminant health by boosting immunity, digestion and reproduction while reducing oxidative stress, methane emissions and disease risks. Key benefits include enhanced milk quality, sperm motility and antioxidant activity. Nutrients, in the form of NPs, can be encased as nanocapsules and transported through the gastrointestinal tract into the bloodstream and then into many body organs, such as the brain, liver, kidney, heart, stomach, intestine and spleen, multiplying the bioavailability of the delivered nutrients (Champion et al. 2007; Iravani et al. 2015). These capsules are proposed to transport the nutrients without any effect on appearance or taste. Encapsulated nanomaterials are combined into fodder as liposomes, micelles and in‐feed bundle systems as recognition markers, biosensors, antimicrobials and shelf‐life extenders (Chen et al. 2006). As for inorganic NPs, minerals have been used widely as NPs such as calcium, magnesium, silicon dioxide and AgNPs in water and animal‐related (Abdelnour et al. 2021). There are many manufacturing methods used for nanomineral fabrication with different physicochemical properties (Abdelnour et al. 2021). Microminerals can be useful for improving health and immunity, digestive system functions, microbiota homeostasis, metabolism and reproductive performance in ruminants (Abdelnour et al. 2021). Additionally, they can be used for producing functional and safe animal products, maybe through eliminating the antibiotic use and increasing concentrations of trace minerals in animal products (meat and milk) required for better human health (Huang et al. 2014; Ezzat Abd et al. 2017; Abdelnour et al. 2021). Male goats that received 0.03 mg/kg of SeNPs had higher final body weight (BW) and average daily gain (ADG) than those that received either sodium selenite or Se‐yeast (Shi et al. 2011). A positive effect has also been found in response to dietary supplementation with 50 mg Zn/kg dry matter (DM), in the form of zinc oxide (ZnO) or ZnONPs, on DM digestibility in Holstein calves (Rajendran et al. 2013). In growing animals, the inclusion of ZnNPs in the diets of lambs enhanced the digestibility and feeding value of the diet, as revealed by the higher feed utilization efficiency observed among the experimental dietary treatments (Mohamed et al. 2015). Kojouri et al. (2019) reported the beneficial impact of SeNP on the BWG of lambs during the second and fourth weeks. Mostly, dietary Zn is included in animal diets in two primary forms: organic (e.g., Zn‐amino acid complexes) and inorganic (such as ZnO and ZnSO_4_). Recently, ZnNPs with size within 1–100 nm is getting more attention for use in the mineral nutrition of livestock to address dietary requirements and to promote animal growth (Swain et al. 2015). The inclusion of ZnNPs (100 and 200 mg/kg) has increased the volatile fatty acids (VFAs), microbial crude protein and degradation of organic matter (OM) at the 6th and 12th h of incubation period under in vitro rumen fermentation conditions (Chen et al. 2011). Similarly, improvement in the microbial biomass production and reduction in methane emanation were recorded with 20 mg of Zn as ZnNPs compared with other higher Zn levels (40 and 60 mg/kg DM) during an in vitro fermentation study (Riyazi et al. 2019). These benefits were also observed in live adult and growing animals. For example, in ewes, adding ZnNPs to their diet improved digestion of DM, OM, nitrogen and crude fibre‐free extract more than larger Zn particles or no supplementation (Mohamed et al. 2017; Hosseini‐Vardanjani et al. 2020). Accordingly, nanominerals have been utilized to modify semen extender properties, aiming to achieve antioxidant and antibacterial effects. Supplementation of bull semen extender with ZnNPs during cryopreservation has been detected to diminish lipid peroxidation and enhance the mitochondrial activity and functionality of sperm plasma membrane in a dose‐dependent manner, without any deleterious effect on motility parameters (Jahanbin et al. 2020). Enrichment of semen extenders with SeNPs at a concentration of 1.0 mg/mL improved post‐thawing sperm quality in Holstein bulls and, thus, the in vivo fertility rate by reducing apoptosis, lipid peroxidation and sperm damage occurring by cryopreservation (Khalil, El‐Harairy et al. 2019). Nanominerals may promote antioxidant activity by inhibiting the free radical's production because of the increased surface area, leading to a higher number of active sites for scavenging an increased number of free radicals (Konvičná et al. 2015). Sheep fed a basal diet containing ZnNPs exhibited a better antioxidant level (Corbo et al. 2015; Kachuee et al. 2019). Supplementation of SeNPs in newborn lambs promoted superoxide dismutase (SOD) levels with a concurrent reduction in thiobarbituric acid–reactive substances (TBARS) values (Mohamed et al. 2017). Nanominerals can also help prevent certain health problems. For example, SeNPs have been shown to protect heart cells from damage caused by ischaemia (Konvičná et al. 2015). Additionally, due to the antibacterial activity of antioxidant nanominerals, some nanominerals, such as ZnONPs, could be helpful in the prevention and curation of some bacterial‐borne diseases such as sub‐clinical mastitis in cows (Rajendran et al. 2013). Petrič et al. (2024) investigated the effect of ZnNPs supplementation on ruminal fermentation, microbiota and histopathology in lambs. The study involved feeding lambs a basal diet supplemented with ZnNPs for 28 and 70 days. Results indicated that ZnNPs supplementation improved feed efficiency, reduced ammonia concentration and increased the activity of hydrolase enzymes in the rumen. Additionally, histological changes in the rumen epithelium were observed, highlighting the positive impact of these NPs on the health of the lambs' rumen. Similarly, Swain et al. (2018) conducted a study to evaluate the efficiency of ZnONPs as a feed supplement on rumen fermentation and nutrient digestibility in goats. They synthesized nano‐sized Zn and divided 24 male goats into four groups, supplementing them with different Zn sources. The results showed that although ZnONPs supplementation improved fibre digestibility and increased rumen pH and protozoa count, it did not affect the overall VFAs profile or other nutrient digestibility (DM, OM, crude protein, ether extract and total carbohydrates) compared to the control group. The study concluded that nano‐Zn can be effectively supplemented at a lower dose than inorganic Zn without negatively impacting rumen fermentation. Furthermore, Hosseini‐Vardanjani et al. (2020) conducted a study to evaluate the effects of dietary supplementation with ZnONPs compared to conventional ZnO on the performance of pregnant Khorasan‐Kurdish ewes. Thirty ewes were divided into three groups, receiving either a control diet, a diet supplemented with ZnO or a diet supplemented with ZnONPs. The results showed that both forms of Zn improved DM intake (DMI), digestibility, ruminal VFAs and total antioxidant capacity, while reducing milk somatic cell count and ruminal ammonia‐N. Ewes fed ZnONPs had higher DMI and DM digestibility than those fed ZnO, and ZnONPs also increased milk Zn concentration without negatively affecting blood serum minerals or enzyme levels in the ewes or their lambs. Overall, ZnONPs supplementation proved beneficial for ewes' health and milk quality. Stress in production animals, particularly in sheep, arises from external stimuli that adversely affect their health and productivity, especially under heat stress (HS) conditions (Al‐Dawood 2017; Collier et al. 2018). HS can lead to infertility through direct effects on the reproductive system or indirectly by reducing feed intake, which alters energy balance and nutrient availability (Ribeiro et al. 2018). Melatonin, a neurohormone with antioxidant properties, plays a crucial role in regulating reproductive functions and has been shown to enhance testosterone secretion and testicular blood flow in rams (Fayez et al. 2024). Additionally, NPs possess unique physicochemical properties that can improve drug stability and bioavailability (Martinelli et al. 2019). In this regard, a study was conducted to assess the impact of melatonin and melatonin NPs on various reproductive parameters in prepubertal Ossimi ram lambs subjected to environmental HS (Fayez et al. 2024). Their findings indicated that both treatments led to significant improvements in testicular echotexture, volume and scrotal circumference, while also enhancing serum levels of oestradiol, nitric oxide and total antioxidant capacity (Fayez et al. 2024). Notably, the nano melatonin treatment demonstrated a more pronounced protective effect against HS compared to standard melatonin, suggesting its potential for improving reproductive efficiency in heat‐stressed farm animals (Fayez et al. 2024). Recent studies highlight the potential benefits of nanominerals, particularly nano‐selenium (N‐Se), as feed supplements to enhance livestock health and fulfil mineral requirements (Budak 2024). N‐Se demonstrates enhanced bioavailability and reduced toxicity compared to conventional selenium forms, serving as an effective immunostimulant with potent antioxidant properties (Budak 2024). Research indicates that N‐Se supplementation can improve liver function by lowering enzyme levels associated with liver damage and enhancing protein metabolism, particularly albumin levels. Additionally, N‐Se positively influences rumen fermentation, promoting a shift in VFAs profiles that supports energy production (Hendawy et al. 2021; Budak 2024). In this context, a study was conducted to explore the impact of nano‐selenium (N‐Se) supplementation on metabolic parameters and rumen fermentation in sheep. The investigation involved twenty female Dorper sheep, with an average weight of 60.1 ± 0.44 kg, divided into two groups, one receiving a Se‐deficient diet and the other supplemented with 3 g/day of N‐Se over 66 days (Budak 2024). The findings revealed that although serum total protein and albumin levels remained unchanged, serum cholesterol, triglycerides, aspartate aminotransferase (AST) and alanine transferase (ALT) levels significantly decreased in the N‐Se group. Furthermore, ruminal ammonia concentration decreased, total VFAs concentration increased and the acetate to propionate ratio declined (Budak 2024). Overall, the study concluded that N‐Se supplementation enhances ruminal fermentation and metabolic health, suggesting its viability as an alternative Se source for sheep (Budak 2024). In a separate study focused on optimizing ruminant performance, the incorporation of magnetic bentonite nanocomposite (MBNC) into diets was examined. The findings revealed that adding MBNC significantly reduced gas production and methane yield while enhancing nutritive parameters and growth performance in Baluchi male lambs. Furthermore, MBNC led to improved digestibility and increased ruminal VFAs, suggesting its efficacy as a dietary supplement for more efficient rumen fermentation and reduced methane emissions (Ibrahimi Khoram Abadi and Heydari 2024). Nannochloropsis species should be prioritized for inclusion in feed formulations due to their suitability for intensive cultivation and their high concentrations of polyunsaturated fatty acids, particularly eicosapentaenoic acid, as well as antioxidants and specific vitamins (El‐Sayed et al. 2024). In this context, the supplementation of Barki ewes with nannochloropsis during the transition period demonstrated significant enhancements in both immune and antioxidant markers in the ewes and their newborn lambs (El‐Sayed et al. 2024). Notably, markers such as NFKB, TNF‐α and various lipogenic factors showed elevated expression in the supplemented group (El‐Sayed et al. 2024). Furthermore, there were improvements in haematological parameters, glucose levels and overall health indicators, along with reduced serum levels of TNF‐α and stillbirth rates in the lambs from supplemented ewes compared to the control group (El‐Sayed et al. 2024). In a study the impact of adding CuO and ZnONPs (0, 10 and 20 mg/mL) on in vitro gas and methane production from alfalfa (*Medicago sativa*) hay was investigated by Palangi et al. (2022). The NPs were characterized through various microscopy techniques, including scanning electron microscopy, Fourier transform infrared (FTIR), energy‐dispersive x‐ray spectroscopy and atomic force microscopy. Results indicated that CuO and ZnONPs significantly influenced ruminal protozoa colonization and methane emissions (Palangi et al. 2022). Although CuO decreased protozoa populations and ruminal fermentation, ZnO enhanced both protozoa growth and gas production, suggesting its beneficial role in rumen fermentation (Palangi et al. 2022). Copper deficiency in dairy cows can lead to numerous health issues, including anaemia, reduced growth and fertility problems (Suttle 2022). This deficiency can manifest in two main forms: primary deficiencies from insufficient dietary Cu and secondary deficiencies caused by dietary antagonists like sulphur and molybdenum (Williams et al. 2024).

To combat these deficiencies, cows are often supplemented with copper in various forms, which can vary in efficacy, especially in the presence of antagonists. Research indicates that using nano copper oxide (NCuO) may improve blood copper levels and overall cow performance more effectively than conventional forms (Shen et al. 2020; Min et al. 2021; Williams et al. 2024). Consequently, a study was conducted to evaluate the copper status in dairy cows fed with NCuO coated with lysine, in comparison to conventional copper oxide (CuO). Utilizing a factorial design, 56 multiparous Holstein‐Friesian cows, averaging 48 days post‐calving, were divided into four treatment groups over a 16‐week period (Williams et al. 2024). The results indicated no significant differences in DMI, milk yield, live weight or body condition score across treatments. However, traditional CuO supplementation led to an increase in somatic cell count compared to NCuO or the treatments with antagonists (Williams et al. 2024). Notably, although plasma molybdenum levels rose in response to antagonists, conventional CuO resulted in decreased liver copper concentration; in contrast, the NCuO with lysine coating increased liver copper concentration even in the presence of dietary antagonists. This research provides novel evidence that NCuO enhances copper bioavailability in dairy cows, without adversely impacting their health or performance (Williams et al. 2024). In a study, the effects of ZnONPs on oxidative stress, inflammation, tight junction integrity and apoptosis in heat‐stressed bovine intestinal epithelial cells (BIECs) were investigated (Li et al. 2023). The research demonstrated that treating BIECs with ZnONPs during HS significantly improved cell viability and reduced apoptosis (Li et al. 2023). Additionally, the treatment enhanced the expression of key antioxidant and anti‐inflammatory genes while promoting tight junction integrity, indicating the potential of ZnONPs to alleviate the negative impacts of HS in dairy cows (Li et al. 2023). Ionophores, such as monensin, are antibiotics that improve ruminal fermentation and feed efficiency in dairy cattle but raise concerns about antimicrobial resistance and environmental impact. This has led to the exploration of natural alternatives (Al Adawi et al. 2024). Gum Arabic (GA), recently approved as a safe feed additive, shows promise in enhancing fermentation, reducing methane emissions and improving animal health (Al Adawi et al. 2024). Meanwhile, montmorillonite clay offers benefits for reducing contaminants and improving ruminal fermentation. Therefore, a study was conducted to evaluate the synergistic effects of GA and nano‐montmorillonite as a novel feed additive. The hypothesis posits that this combination can enhance ruminal fermentation and overall health in dairy cows when compared to traditional ionophores (Al Adawi et al. 2024). In this study, the physicochemical properties and effects of Arabic gum–nano montmorillonite (AGNM) as a feed additive were evaluated against the conventional ionophore monensin in Holstein dairy cows. Using a 4 × 4 Latin square design, four mid‐lactation cows were assigned to different dietary treatments: a control diet, a monensin‐supplemented diet and AGNM diets at low (1.5 g/kg) and high (3 g/kg) levels (Al Adawi et al. 2024). Results showed that high AGNM supplementation significantly enhanced the concentrations of total VFAs and digestibility of fibre and OM and reduced methane production (Al Adawi et al. 2024). Additionally, it improved blood high‐density lipoprotein levels while decreasing several metabolites such as creatinine and cholesterol. Both monensin and high AGNM increased feed efficiency, but neither affected milk composition or energy status. The findings support the potential of AGNM as a natural alternative to monensin, improving ruminal fermentation and nutrient use in dairy cows (Al Adawi et al. 2024). ZnONPs are essential metal oxide nanomaterials with significant properties such as stability, catalytic activity and antibacterial effects (Quintão et al. 2024). In vitro embryos, although beneficial for improving fertility and offspring health, face challenges such as oxidative stress due to excessive ROS generation during cultivation (Quintão et al. 2024). Zn plays a vital role in reducing ROS levels and enhancing embryonic development. In this regard, a study was conducted to explore the effects of incorporating ZnONPs into in vitro maturation (IVM) media on the developmental, cryosurvival and metabolic characteristics of bovine embryos (Quintão et al. 2024). Three different concentrations of ZnONPs (0, 1.0 and 1.5 µg/mL) were tested (Quintão et al. 2024). The findings revealed that the addition of ZnONPs at a concentration of 1.0 µg/mL significantly increased the number of embryonic cells while simultaneously reducing the production of ROS (Quintão et al. 2024). Importantly, ZnONPs did not affect embryonic development, cryosurvival rates or mitochondrial viability (Quintão et al. 2024). These results indicate that ZnONPs possess antioxidant properties and are compatible with bovine oocytes, suggesting their potential as beneficial supplements to IVM media, thereby enhancing the efficacy of assisted reproductive techniques (Quintão et al. 2024). Artificial insemination is an ancient biotechnological method crucial for modern animal breeding. Effective sperm preservation requires minimizing oxidative stress caused by ROS, which can impair sperm function (Vishwanath 2003). Berberine, a natural compound with low solubility and bioavailability, is combined with eco‐friendly SeNPs to improve antioxidant properties during sperm preservation (Mullauer et al. 2011; Piri et al. 2024). In this context, a study was conducted to evaluate the effects of nano‐berberine and Se‐loaded berberine NPs on the characteristics of goat sperm following cryopreservation. ROS pose a significant threat to sperm integrity, with antioxidants like berberine, an alkaloid derived from sources such as *Berberis vulgaris*, *Curcuma longa* and *Ergon grape* serving as crucial protectants against oxidative damage, despite berberine's limited bioavailability (Piri et al. 2024). To enhance its efficacy, berberine was incorporated into SeNPs synthesized using barberry extract, and the NPs were characterized through techniques such as EDX, UV–visible spectroscopy, FE‐SEM, zeta potential and FTIR. The experimental results demonstrated statistically significant improvements in motility, viability, DNA integrity, membrane integrity, ROS levels, lipid peroxidation, in vitro fertilization rates and developmental outcomes of treated oocytes compared to the control group (Piri et al. 2024). Notably, the combination of berberine infused into SeNPs yielded the most pronounced benefits for sperm quality post‐thawing (Piri et al. 2024). In another study aimed at assessing the impact of oral SeNPs supplementation on various serum biomarkers and BWG, 12 newborn lambs were evaluated (Kojouri et al. 2019). The lambs were divided into two groups, with one receiving SeNPs and the other receiving a control treatment of distilled water over 7 consecutive days. Findings revealed that SeNP supplementation significantly elevated serum Se levels while reducing copper and Zn concentrations (Kojouri et al. 2019). Notably, by Day 14, TBARS levels were elevated in the treated group, but both SOD and TBARS levels showed favourable changes by Day 28 (Kojouri et al. 2019). Additionally, SeNPs contributed to improved BWG by the 14th and 28th days, indicating their beneficial role in enhancing antioxidant activity and growth rates in neonatal lambs (Kojouri et al. 2019). Furthermore, a study was conducted to evaluate the effects of dietary supplementation with ZnONPs versus traditional ZnO on various health and performance metrics in pregnant Khorasan‐Kurdish ewes (Hosseini‐Vardanjani et al. 2020). The experiment involved 30 ewes divided into three groups, receiving control, ZnO or ZnONPs diets during the pre‐ and post‐partum periods (Hosseini‐Vardanjani et al. 2020). Results indicated that both forms of Zn supplementation improved DMI, digestibility, ruminal VFAs and total antioxidant capacity while reducing somatic cell counts. Notably, ewes receiving ZnONPs demonstrated significantly enhanced DMI and post‐partum digestibility, alongside higher concentrations of Zn in milk, without detrimental effects on blood serum mineral levels or enzyme activity in both ewes and their lambs (Hosseini‐Vardanjani et al. 2020). This suggests that ZnONPs may be a beneficial alternative to ZnO for enhancing ewe performance and health markers (Hosseini‐Vardanjani et al. 2020). Animal productivity is adversely affected by environmental factors like HS, which leads to reduced feed intake and increased respiration rates, particularly in sheep (Hung et al. 2021). Effective heat dissipation in sheep relies on evaporation rather than sweating. Dietary chromium (Cr) supplementation has shown potential benefits in improving feed intake and physiological responses during stress conditions, though results are inconsistent (Hung et al. 2021). Recent studies suggest that chromium NPs (CrNPs) may enhance digestibility and improve growth rates under HS (Hung et al. 2021). In this regard, the effects of dietary nano chromium picolinate (nCrPic) on sheep under HS were evaluated (Hung et al. 2021). Thirty‐six Merino × Poll cross‐bred sheep were subjected to three dietary treatments (0, 400 and 800 µg nCrPic/kg) over an 8‐week period, followed by exposure to thermo‐neutral (TN) and HS conditions for 3 weeks. Although nCrPic did not affect growth performance during TN conditions, it significantly increased average daily feed intake (ADFI) and ADG under HS. Furthermore, sheep receiving nCrPic experienced lower rectal temperatures and respiration rates during peak HS periods (Hung et al. 2021). These findings suggest that nCrPic can alleviate some of the adverse effects of HS in sheep by maintaining ADFI and decreasing physiological stress responses (Hung et al. 2021).

### 7.2 Impacts of NPs in Camel

**FIGURE 3:**
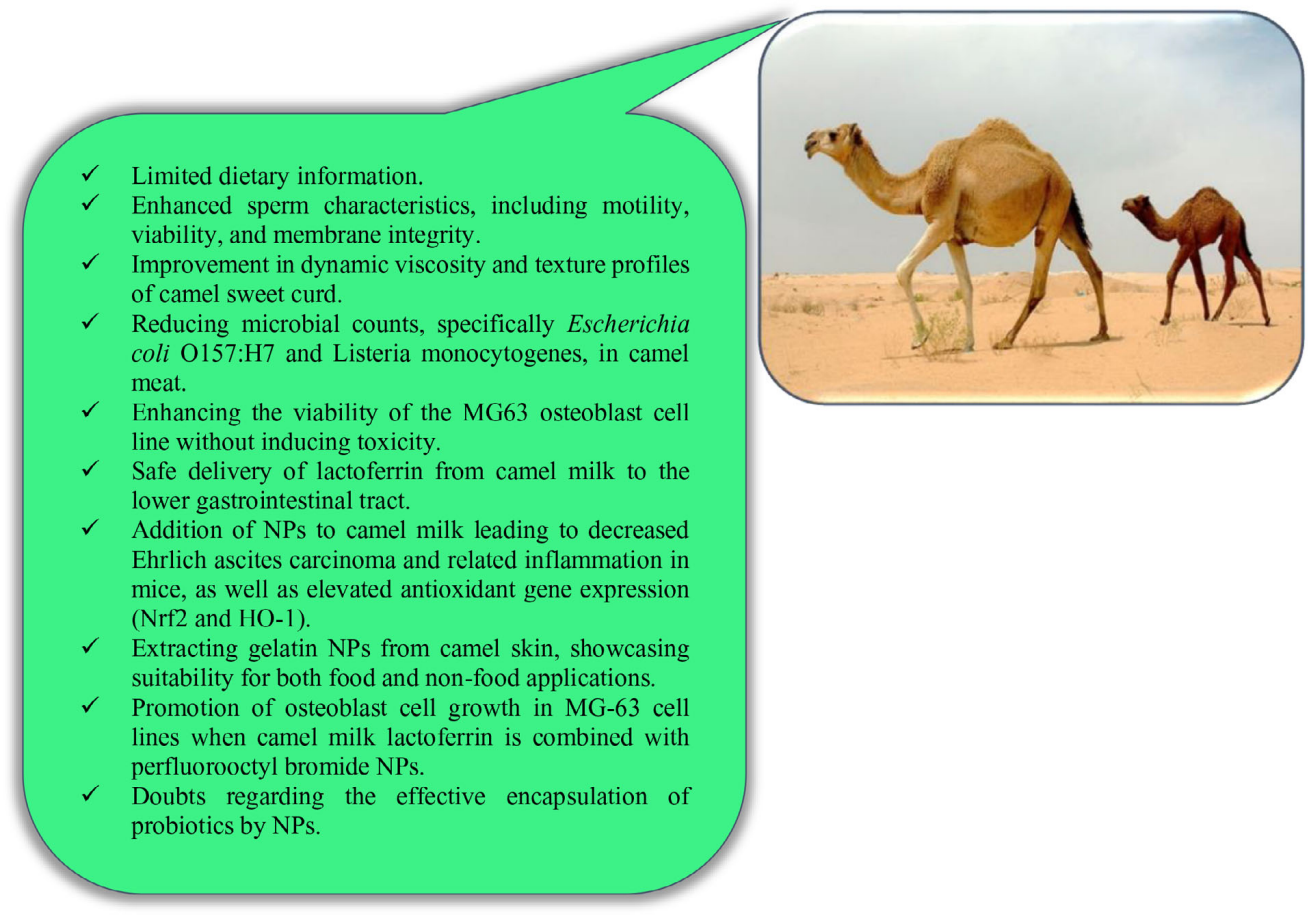
Nanoparticles (NPs) for camel use.

There is limited information available regarding the use of NPs in camel nutrition; however, research has been conducted to some extent on other practical aspects of NPs for camels. A study was conducted to investigate the impact of antioxidant supplementation, SeNPs and ZnNPs and storage duration at low temperatures on the quality of epididymal camel spermatozoa (Shahin et al. 2021). Testes were collected post‐slaughter and maintained at 4°C until processing (Shahin et al. 2021). Spermatozoa were extracted, diluted with SHOTOR extender and supplemented with various antioxidants and then stored at 4°C for intervals of 2, 48, 96 and 144 h. The results indicated that storage time significantly influenced sperm motility, viability, membrane integrity and the presence of cytoplasmic droplets. Notably, the addition of antioxidants such as nano‐Se and Zn significantly enhanced sperm characteristics compared to the control group. Overall, the incorporation of nano‐sized minerals and vitamins effectively preserved sperm quality during storage for up to 144 h (Shahin et al. 2021). The incorporation of guadar cereal into camel milk has spurred the demand for innovative, nutritious weaning food formulas. In this context, a study aimed to develop both wet and dry NP weaning formulas using various combinations of guadar flour, camel sweet curd and whey. The pH values of the mixtures were between 5.92 and 6.43, with protein content ranging from 5.36% to 10.72%. Particle size increased with the addition of sweet curd, measuring between 60.7 and 77.56 nm. Dynamic viscosity and texture profiles significantly improved with guadar NP flour (Desouky and Salama 2020). All formulas received positive sensory evaluations, with the T1 formula, which had a higher proportion of guadar flour, being rated the best (Desouky and Salama 2020). Overall, this new weaning food represents a promising commercial opportunity to fill the nutritional gap for young children in arid and semi‐arid regions (Desouky and Salama 2020). The application of ZnNPs as a protective barrier against bacteria in the food industry is widely regarded as advantageous. Furthermore, the incorporation of antioxidant and polyphenolic compounds found in specific herbs can amplify this protective effect (Gharehyakheh 2023). In this regard, ZnNPs were synthesized through a green approach utilizing *Anethum graveolens* leaf extract (Gharehyakheh 2023). An illustration of NPs for camel use is shown in Figure 3. The figure illustrates key findings about NPs’ benefits for camel‐derived products. These include improved sperm quality regarding motility and viability, better dairy texture characteristics, reduced pathogen levels in meat such as *Escherichia coli* and Listeria, safe lactoferrin (LF) delivery and demonstrated anticancer and anti‐inflammatory effects in mice studies. Researchers found NPs extracted from camel skin have potential for both food and non‐food applications. Additionally, camel LF combined with perfluorooctyl bromide (PFOB) NPs shows osteoblast growth promotion. However, limitations remain regarding the effectiveness of probiotic encapsulation methods. The antibacterial efficacy of these NPs was evaluated against *E. coli* O157:H7 and *Listeria monocytogenes* in camel meat over 20 days, using concentrations ranging from 0% to 1% for three protective agents: *A. graveolens* L., ZnNPs and Zn (NO_3_)_2_. The findings revealed that ZnNPs at 1% concentration were the most effective, reducing microbial counts significantly (Gharehyakheh 2023). Additionally, higher concentrations of all substances provided enhanced antibacterial protection, whereas parameters such as peroxide value, pH, protein carbonyl content, total volatile basic nitrogen, and TBARS were maintained at lower levels compared to the control. Cytotoxicity tests confirmed that both *A. graveolens* L. and ZnNPs were non‐toxic. Overall, the research underscores the potential of integrating natural materials with chemical methods to improve food safety and quality (Gharehyakheh 2023). *L. monocytogenes* is a noteworthy foodborne pathogen associated with outbreaks linked to soft cheeses and meat products. This underscores the importance of implementing alternative strategies to manage its proliferation in food items (Korany et al. 2024). Therefore, a study was conducted on eco‐friendly ZnONPs synthesized using extracts from *Rosmarinus officinalis*, *Punica granatum* and *Origanum marjoram* (Korany et al. 2024). The results indicated that the ZnONPs containing *O. marjoram* exhibited the highest antimicrobial efficacy against *L. monocytogenes*, leading to their application in the preparation of gelatin‐based bionanocomposite coatings (Korany et al. 2024). These coatings were effective in reducing *L. monocytogenes* counts in Talaga cheese and camel meat, as well as controlling pH changes and improving sensory characteristics, ultimately extending the shelf life of these food products (Korany et al. 2024). A study was conducted to investigate the effects of LF extracted from camel milk and its interaction with calcium alginate (Alg) hydrogel NPs on the MG63 osteoblast cell line (Reyhani et al. 2021). The LF was purified using HPLC and SDS–PAGE techniques, and subsequent interactions with Alg were analysed at concentrations of 0.2% and 0.5% across pH levels 6, 7 and 8. Spectroscopic methods, zeta potential and dynamic light scattering particle size analysis revealed a substantial reduction in zeta potential, indicating the formation of LF‐Alg complexes, with a notable loading efficiency of 89.9% (Reyhani et al. 2021). The MTT (3‐(4,5‐dimethylthiazol‐2‐yl)‐2,5‐diphenyltetrazolium bromide) assay confirmed that LF‐Alg particles enhance MG63 cell viability without inducing toxicity, with optimal interaction observed at pH 8, thereby promoting cell growth effectively (Reyhani et al. 2021). In a similar study conducted by some researchers, it was investigated how alginate concentration (0.2 and 0.5 w/w%) and encapsulation techniques (thermal vs. non‐thermal treatment) affected the encapsulation efficiency, zeta potential, particle size and LF release from nanocapsules (Raei et al. 2015). The isolated LF from camel milk was nano‐encapsulated using alginate nanocapsules. Notably, lactoperoxidase was found in NaCl fractions of 0.8 and 0.9 M, disqualifying them for further testing (Raei et al. 2015). The findings showed that higher alginate concentrations enhanced encapsulation efficiency, with thermal treatment yielding nearly 100% efficiency and smaller particle sizes (mostly < 100 nm). Interestingly, LF exhibited no release during the first 30 min at both pH levels (2 and 7), suggesting that it remains intact under stomach conditions, allowing safe delivery to the lower gastrointestinal tract (Raei et al. 2015). Osteoporosis is a prevalent condition that is characterized by reduced bone density, which poses significant health risks. Recent studies have shown that LF promotes osteogenesis (Shadan et al. 2017). PFOB serves as a neutral particle utilized as a drug carrier. Hence, in a study, LF derived from camel milk was isolated. The PFOB nano‐emulsion particles (PFOB‐NEPs) were prepared using a method that involves oil‐in‐water emulsification (Shadan et al. 2017). The properties of the NPs through zeta potential measurements and tryptophan fluorescence spectroscopy, both before and after loading with LF, were assessed (Shadan et al. 2017). The findings indicated that LF from camel milk significantly stimulates the proliferation of osteoblasts in MG‐63 cell lines. Furthermore, it is feasible to encapsulate LF within PFOB‐NEPs, demonstrating that these NPs can effectively serve as carriers for LF (Shadan et al. 2017). Enhancing the quality of life for cancer patients is essential, and this can be achieved through the development of treatments that effectively combat cancer while minimizing adverse effects (Tawfic et al. 2024).

Hence, a study investigated the effects of CNPs and camel milk exosomes (CMEs), both individually and alongside Sorafenib (SOR), on mice with Ehrlich ascites carcinoma (EAC) (Tawfic et al. 2024). The study also aimed to determine if this combination could improve liver damage associated with EAC. Liver function and oxidative stress levels were measured spectrophotometrically, inflammatory cytokine levels were assessed using enzyme‐linked immunosorbent assay, and gene expression was analysed through real‐time polymerase chain reaction (Tawfic et al. 2024). Treatment with CNPs, CMEs and SOR significantly reduced tumour burden in mice with EAC. Indicators of this reduction included decreased ascitic fluid volume, enhanced apoptosis (elevated p53, Bax and caspase 3 and lowered Bcl2), increased ROS, diminished migration (higher matrix metalloproteinase 9 and lower tissue inhibitor of metalloproteinases‐1) and reduced angiogenesis (lower vascular endothelial growth factor) (Tawfic et al. 2024). Additionally, the treatments alleviated EAC‐induced liver damage, evidenced by improved liver function (normal ALT, AST, ALP and albumin levels), restored redox balance (reduced malondialdehyde [MDA] and increased antioxidant enzyme activities), elevated antioxidant gene expression (Nrf2 and HO‐1), reduced inflammation (lower IL1β and TNFα levels) and improved liver structure (Tawfic et al. 2024). Notably, mice treated with SOR and CNPs exhibited the greatest benefits, followed by those receiving SOR and CMEs. In conclusion, CNPs and CMEs significantly enhanced the anticancer effects of SOR and mitigated liver injuries caused by EAC (Tawfic et al. 2024). Gelatin is a protein substance that is primarily produced through the partial hydrolysis of collagen, which is sourced from the skin, bones and connective tissues of terrestrial mammals, as well as from fish and poultry (Ahmed et al. 2020). This versatile ingredient is widely employed in various applications to improve elasticity, increase thickness and facilitate emulsification (Ahmed et al. 2020). In this context, a study was conducted with the aim of extracting gelatin from camel skin as an alternative source through a series of processing steps (Ahmed et al. 2020). The analysis of the gelatin NPs (GNPs) derived from camel skin, along with their functional properties, demonstrated their strong suitability for both food and non‐food applications, highlighting their potential for use in the expanding global halal food market (Ahmed et al. 2020). Research indicates that nano biomolecules extracted from the biological fluids of camels possess numerous therapeutic properties beneficial to humans (Atiroğlu et al. 2024). Recent research investigated the use of innovative starch‐based NPs for the encapsulation of probiotic bacteria derived from camel milk (Ahmad et al. 2019). It was observed that encapsulation in native starch provided superior cell viability and thermal protection compared to other methods (Ahmad et al. 2019). In contrast, probiotic cells that were nano‐encapsulated exhibited reduced viability when compared to those encapsulated within native starch (Ahmad et al. 2019). Furthermore, it was found that the nano‐starch particles failed to provide adequate thermal protection for the probiotic cells. Consequently, this study suggests that NPs may not be the ideal choice for effectively encapsulating probiotics (Ahmad et al. 2019).

### 7.3 Impacts of NPs in Chicken

**FIGURE 4:**
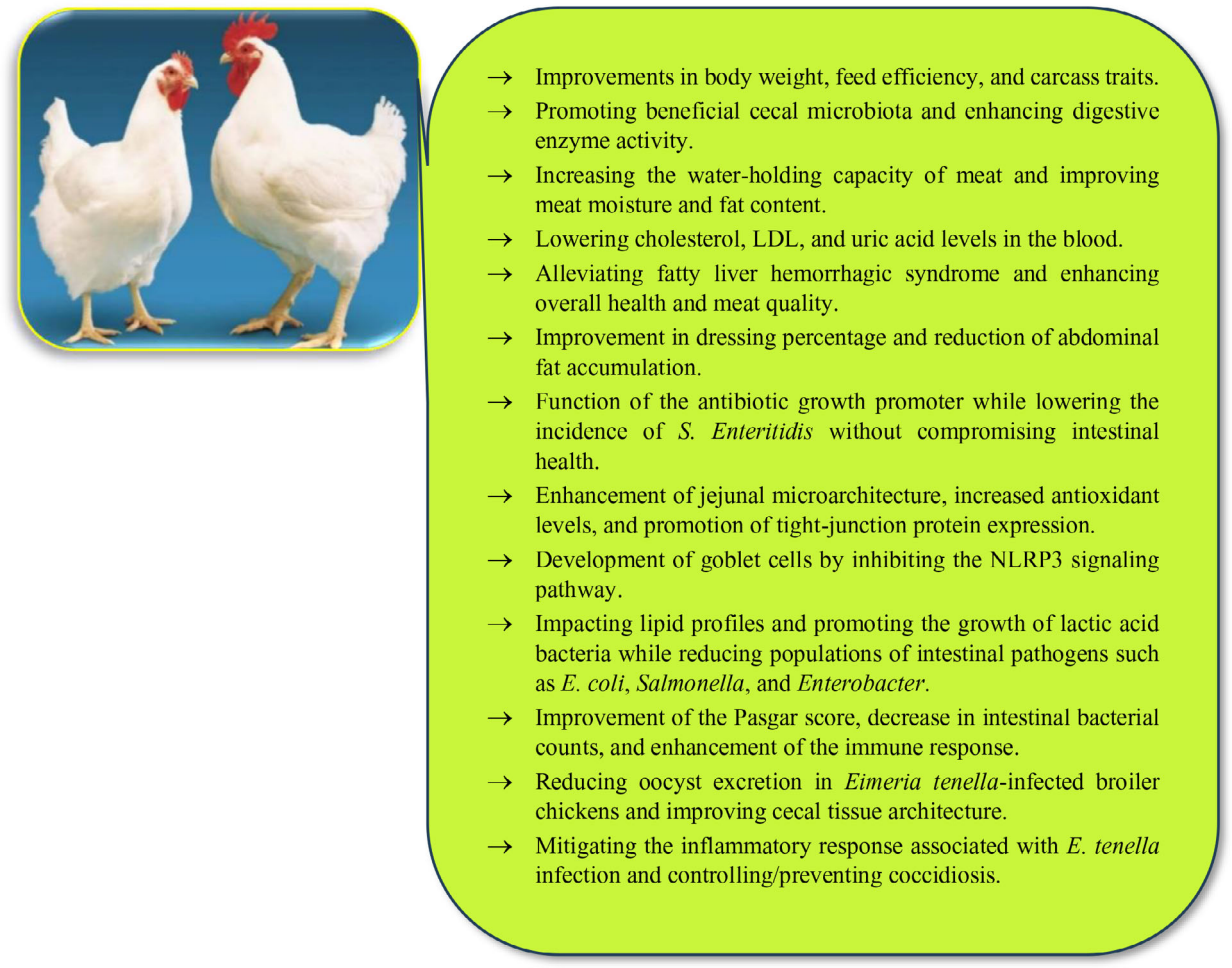
Main effects of nanoparticles in chicken. LDL, low‐density lipoprotein.

ZnONPs have garnered significant attention due to their unique properties. These NPs, synthesized through nanotechnology and measuring between 1 and 100 nm, serve as a bioavailable form of Zn, a crucial trace element for numerous biological and physiological functions. Research has demonstrated that ZnONPs exhibit effective immunomodulatory, growth‐promoting and antimicrobial effects in poultry, enhancing productivity and welfare while potentially minimizing environmental impact compared to traditional Zn sources. However, they may also pose toxicological risks, which vary based on factors such as size, shape, concentration and exposure routes (Fatima et al. 2023). An illustration of the main effects of NPs in chicken is presented in Figure 4. As clearly illustrated in the figure, NPs significantly enhance poultry performance by improving growth metrics (BW, feed efficiency), carcass quality and meat properties (water‐holding capacity, fat content). They optimize gut health through microbiota modulation (increased probiotics, reduced pathogens like *E. coli/Salmonella*), enhanced digestive enzymes and improved intestinal integrity (tight junctions, goblet cell development). Blood parameters (cholesterol, low‐density lipoprotein) and liver health show marked improvement, whereas antioxidant and immune responses are boosted. Notably, NPs effectively control infections (*Salmonella enteritidis*, *Eimeria tenella*) by reducing pathogen loads, oocyst excretion and inflammation, demonstrating dual growth‐promoting and disease‐preventing roles without compromising intestinal health. AL‐Ruwad et al. (2024) investigated the effects of copper NPs (CuNPs) on various parameters in broiler chickens, including growth performance, carcass characteristics and health metrics. A total of 300 Arbor Acre chicks were divided into five groups, with one receiving a control diet and the others receiving diets supplemented with CuNPs at varying concentrations (5, 10, 15 and 20 mg/kg) (AL‐Ruwad et al. 2024). Results indicated significant improvements in BW, feed efficiency and carcass traits, particularly at the 15 mg/kg level. Additionally, CuNPs enhanced immune function, antioxidant status and lipid profiles, while also promoting beneficial cecal microbiota and digestive enzyme activity. Overall, CuNPs supplementation proved beneficial for the performance and health of broiler chicks (AL‐Ruwad et al. 2024). Likewise, the results of a study showed that broilers receiving a supplementation of 12 mg/kg of CuNPs demonstrated improved average weight and feed conversion ratio (FCR) when compared to the other groups (Ahmed et al. 2024). Although feed intake and carcass characteristics remained unaffected, the metabolizability of crude fat was significantly greater in this group (Ahmed et al. 2024). Additionally, higher concentrations of catalase and SOD were observed in the CuNP‐supplemented groups, with the water‐holding capacity of meat also notably increased in the 12 mg/kg group (Ahmed et al. 2024). A separate study evaluated how different dietary levels of organic ZnNPs influence growth performance, carcass characteristics, meat quality and blood parameters in broiler chickens (Ashour et al. 2024). Supplementation with 0.4 mg/kg ZnNPs significantly increased BW and BWG, improved daily feed intake and enhanced certain carcass characteristics compared to the control group (Ashour et al. 2024). Additionally, this treatment resulted in lower cholesterol, low‐density lipoprotein and uric acid levels in the blood, as well as improved meat moisture and fat content and reduced counts of total yeast/mould and *E. coli*, demonstrating enhanced health and meat quality in the broilers (Ashour et al. 2024). Poly‐dihydromyricetin‐fused ZnNPs have demonstrated potential in alleviating fatty liver haemorrhagic syndrome by enhancing antioxidant capacity, modulating liver lipid metabolism and supporting intestinal health (Yang et al. 2024). Similarly, broiler chickens receiving diets supplemented with nano‐Fe oxide at concentrations of 20 and 40 mg/kg exhibited significant improvements in growth performance, meat quality, tissue iron content, dressing percentage and reduced abdominal fat accumulation (Almeldin et al. 2024). Studies investigating the use of CuNPs in broiler diets have assessed their potential as alternatives to antibiotic growth promoters (AGPs) (Patrin Pontin et al. 2024). The results revealed that CuNP significantly lower the incidence of *S. enteritidis* without compromising the intestinal health of the chickens, thereby reinforcing the prospect of CuNP as an effective antimicrobial agent in poultry production (Patrin Pontin et al. 2024). Another study demonstrated that maternal supplementation with nano‐Se enhanced the jejunal microarchitecture, increased antioxidant levels and promoted the expression of tight‐junction proteins in chicken offspring (Chen et al. 2024). Furthermore, this supplementation facilitated the development of goblet cells by inhibiting the NLRP3 signalling pathway (Chen et al. 2024). Additionally, biological nano‐selenium (BNSe) positively impacted lipid profiles and promoted the growth of lactic acid bacteria, while concurrently reducing the populations of various intestinal pathogens such as *E. coli*, *Salmonella* and *Enterobacter* (Reda et al. 2023). Sherif et al. (2024) revealed that the dietary supplementation of *Chlorella vulgaris* dried powder with ZnONPs and SeNPs significantly enhanced the growth parameters of broilers, including final BW and feed intake.

Notable improvements were observed in FCR and intestinal morphology, alongside increases in antioxidant enzyme activities and the expression of growth‐related genes. Additionally, a positive effect on immune response was indicated by an increased phagocytic index and alterations in cytokine gene expression. Furthermore, they noted that the combination of CV with ZnONPs or SeNPs proved to be beneficial for growth and antioxidant capacity in broilers (Sherif et al. 2024). In a study, it was found that the in ovo injection of copper oxide NPs (CuO‐NPs) did not significantly impact hatchability traits or chick quality in newly hatched broiler chicks (Issa et al. 2024). Specifically, hatchability, chick yield percentage, yolk‐free body mass, chick length, shank length and relative weights of heart, gizzard and intestine remained unchanged across all treatment groups. Notably, the Pasgar score showed a slight improvement in the CuO–NP groups compared to the negative control and positive control groups (Issa et al. 2024). The administration of 60 ppm CuO‐NPs resulted in a significant increase in intestinal length. Both doses of CuO‐NPs also raised the concentration of copper ions in hepatic tissue, and varying degrees of tissue damage were observed in the livers of chicks subjected to either low or high doses of CuO‐NPs. They ultimately observed that although the in ovo injection of CuO‐NPs had a positive effect on chick appearance, concerns about liver tissue damage indicate potential risks associated with their use in in ovo applications (Issa et al. 2024). Furthermore, a study demonstrated that in ovo administration of CuNPs and copper sulphate (CuSO_4_) significantly enhances broiler chicken performance (Mroczek‐Sosnowska et al. 2015). By the end of the rearing period on Day 42, BW was notably greater in the nano‐Cu (2206 g) and CuSO_4_ (2402 g) groups compared to the control group, which weighed only 2000 g. Both treatment groups exhibited a significantly reduced feed conversion rate and mortality, alongside a higher percentage of breast and leg muscle in the carcass relative to the control (Mroczek‐Sosnowska et al. 2015). The in ovo application of copper colloids may facilitate effective penetration into embryonic tissue, resulting in sustained positive effects on growth postnatally. This approach presents a promising alternative to the conventional use of copper as a feed additive (Mroczek‐Sosnowska et al. 2015). Vitamin E (VE) serves as a critical chain‐breaking antioxidant within cells and is regarded as the central component of the antioxidant defence system, effectively mitigating the risk of cellular and tissue damage caused by free radical‐induced lipid peroxidation (Zhou et al. 2024). However, both natural and synthetic forms of VE are prone to degradation at different phases of feed processing, production and storage, which complicates their effectiveness as feed additives. Significant efforts have been undertaken to improve the integration and utilization of VE in food products, focusing on the development of efficient delivery systems that protect VE from chemical degradation and enhance its bioavailability when consumed (Yang and McClements 2013). Nano‐emulsions have emerged as a promising solution in food applications due to their high oral bioavailability and the potential to create sturdy and clear drug delivery systems (Golfomitsou et al. 2018). Additionally, the susceptibility of both natural and synthetic VE to degradation during various feed processing stages presents significant challenges for their application as nutritional supplements (Zhou et al. 2024). Nanotechnology enhances the absorption and retention of VE in the bloodstream of broilers, thereby optimizing its efficacy and boosting the antioxidant capabilities of these birds (Zhou et al. 2024). In another study, the impact of thyme, ginger and their NPs as alternatives to AGPs on the performance, carcass traits, meat quality and gut health of broiler chickens was investigated (Hassan et al. 2024). A total of 270 1‐day‐old chicks were divided into six groups, with varying diets over 35 days: a control group, an AGPs group, groups receiving thyme and ginger (1.0% each) and groups with nano‐thyme and nano‐ginger (0.10% each). Results indicated that both thyme and ginger, along with their nano‐forms, enhanced BW, BWG and feed conversion rates, with nano‐ginger showing the most significant effects (Hassan et al. 2024). Notably, these phytogenic additives improved meat quality attributes and decreased intestinal bacterial counts, positioning them as effective AGPs alternatives that mitigate health risks and reduce feeding costs through reduced dosages (Hassan et al. 2024). A study was performed to assess the effects of nanomanganese on the antioxidant status, haematological changes and protein docking in broiler chickens (Helbawi et al. 2024). Results indicated that administering nanomanganese at concentrations of 66 and 72 mg/kg yielded beneficial outcomes on productivity, antioxidant levels and haematological parameters in the chicks. Specifically, the inclusion of 72 mg of manganese in the form of MnO_2_ NPs demonstrated superior effects across the majority of the evaluated parameters, exerting no adverse impacts on the broiler chicks (Helbawi et al. 2024). The study demonstrated that ultrasonicated *R. officinalis* ethanolic extract (UROEE), chitosan‐loaded NPs (CsNPs) and their combination (UROEE‐CsNPs) effectively reduced oocyst excretion in *E. tenella*‐infected broiler chickens and improved cecal tissue architecture (Kasem et al. 2024). Specifically, there was a notable decrease in the protein expression of CD4+ and CD8+ T lymphocytes. Primary infection resulted in elevated levels of pro‐inflammatory cytokines (IFN‐γ, IL‐1β, IL‐6) and anti‐inflammatory cytokines (TGF‐β4), whereas secondary infection led to decreased expression of these cytokines (Kasem et al. 2024). The administration of UROEE, CsNPs and UROEE‐CsNPs as dietary additives and therapeutic treatments mitigated the inflammatory response associated with *E. tenella* infection. These findings suggest that these agents could be utilized to enhance strategies for controlling and preventing coccidiosis in poultry (Kasem et al. 2024). A study assessed the effects of CNPs on the performance and health of broiler chickens (Adil et al. 2024). The results indicated that supplementation with 0.4 g CNPs/kg significantly enhanced BW, BWG, feed efficiency and nutrient digestibility while lowering serum cholesterol and microbial load (Adil et al. 2024). Additionally, birds receiving this dosage exhibited improved meat quality characteristics, suggesting that 0.4 g/kg of CNPs is a beneficial additive for broiler diets (Adil et al. 2024). Although NPs offer various benefits for poultry, their reported toxic effects raise safety concerns. For instance, dietary CuO‐NPs exhibit dose‐dependent toxicity in chickens, triggering oxidative stress, DNA damage and organ dysfunction, along with impaired growth and immune response (Morsy et al. 2021). Furthermore, another study revealed that titanium dioxide NPs induce toxic effects in chicken embryos even at low doses (150–300 µg/mL) (Islam et al. 2024). Exposure caused reduced BW, liver damage, immune tissue alterations and suppressed expression of critical immune genes (AvBD9, IL6, IL8L2) (Islam et al. 2024). These findings warrant caution in agricultural NPs use.

### 7.4 Dietary Impacts of NPs in Quail

**FIGURE 5:**
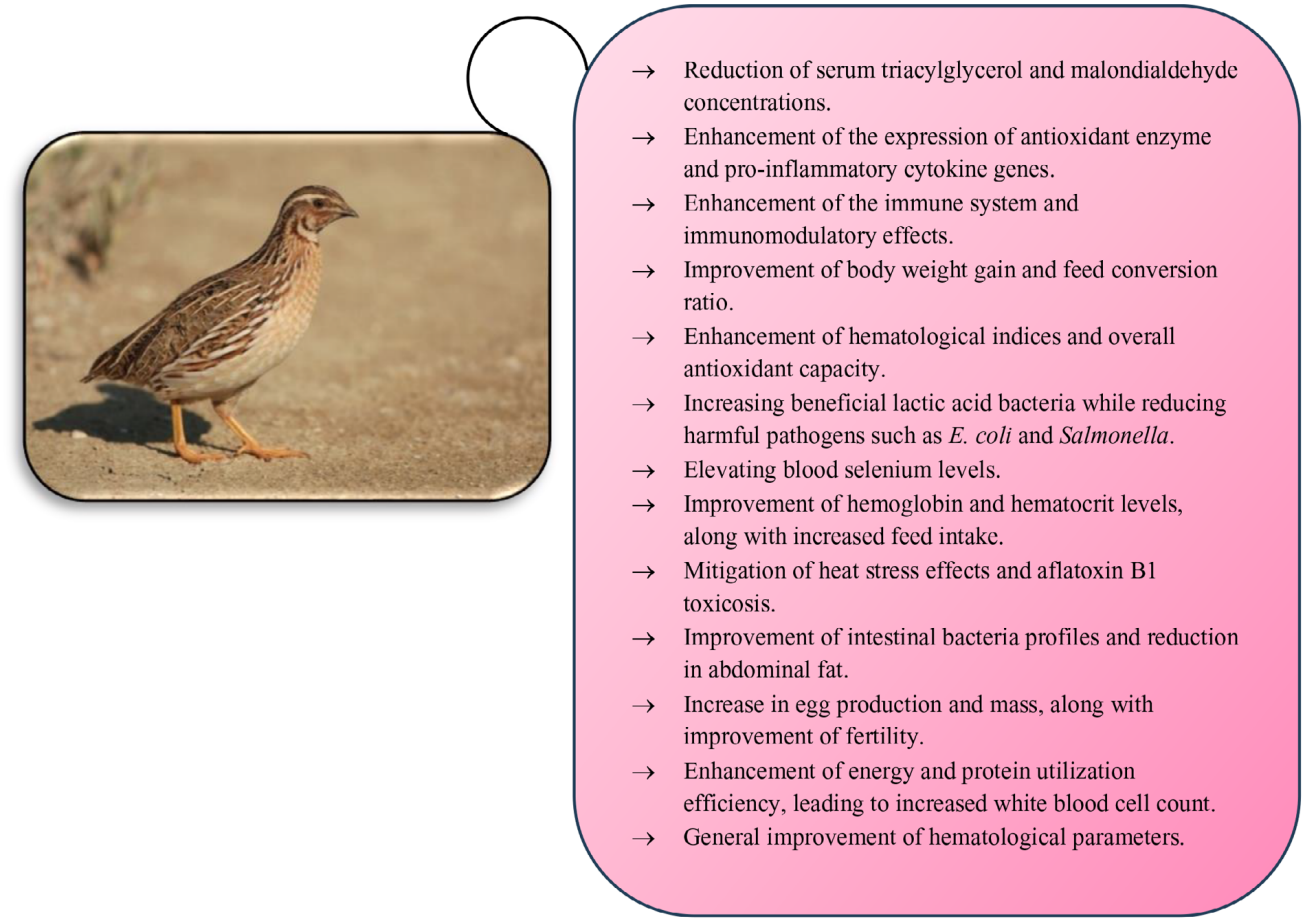
Main effects of nanoparticles in quail.

Zn is a crucial trace element for poultry, playing a significant role in enhancing the growth performance, skeletal muscle development and immune function of broiler chickens and quail (Zhang et al. 2018; El‐Bahr et al. 2020). Due to the poor bioavailability of Zn, it is essential to meet the animals’ requirements by increasing the concentration of inorganic Zn approximately 20–30 times above the optimal levels (Bratz et al. 2013; El‐Bahr et al. 2020). However, this approach can be costly, may lead to increased Zn residues in chicken manure and could negatively impact the digestibility of other trace elements, such as copper, iron and cadmium (Cd) (El‐Bahr et al. 2020). Consequently, enhancing the bioavailability of Zn is a vital prerequisite for addressing these challenges. An illustration of the main effects of NPs in quail is presented in Figure 5. As described in the figure, NPs significantly enhance quail health and productivity by improving metabolic markers (reduced triacylglycerol, MDA) and boosting antioxidant capacity through upregulated enzyme expression. They strengthen immunity via elevated cytokine genes, increased white blood cells and optimized haematological indices (haemoglobin, haematocrit). NPs enhance growth performance (BW, FCR) and gut health by promoting lactic acid bacteria while suppressing pathogens (*E. coli*, *Salmonella*). Additionally, they mitigate stressors (heat, aflatoxin B1) and improve reproductive outcomes (egg production, fertility) alongside better nutrient utilization and reduced abdominal fat. There has been a growing interest in replacing bulk inorganic trace elements with NPs in recent years. In this regard, a study investigated the effects of varying dietary levels of ZnONPs on serum biomarkers, lipid peroxidation and gene expression of antioxidant enzymes and cytokines in Japanese quail (El‐Bahr et al. 2020). Eighty quails were divided into four groups, with the experimental groups receiving 15, 30 or 60 mg/kg of ZnONPs over 60 days (El‐Bahr et al. 2020). Results indicated that ZnONPs at 30 and 60 mg/kg significantly reduced serum triacylglycerol levels and MDA concentrations without affecting liver enzyme activity or total protein levels. Additionally, higher doses of ZnONPs enhanced the expression of antioxidant enzyme and pro‐inflammatory cytokine genes, suggesting that dietary ZnONPs, particularly at 60 mg/kg, can potentially enhance the antioxidant and immune response in Japanese quails (El‐Bahr et al. 2020). SeNPs have been effectively synthesized using various green nanotechnology methods, including the incorporation of plant extracts, as indicated by Zambonino et al. (2021) and Medina Cruz et al. (2018). The green synthesis approach provides an environment‐friendly alternative for NP production, employing both unicellular and multicellular organisms, such as yeast, fungi, bacteria, plant tissues and algae, which serve as natural reducing and stabilizing agents (Malyugina et al. 2021). Numerous studies have shown that the form and dosage of Se in the diet can significantly influence growth performance, antioxidative properties, immune response and meat quality characteristics in broiler chickens and quail. In this regard, a study demonstrated that Japanese quails receiving a dosage of 0.2 mg/kg of SeNPs experienced enhanced BWG and an improved FCR (Naz, Bibi et al. 2024). In contrast, a higher dosage of 0.4 mg/kg resulted in diminished growth performance, which was associated with considerable histological damage and genotoxic effects (Naz, Bibi et al. 2024). Furthermore, in this context, different concentrations of nano‐Se in conjunction with environmental temperature may influence the growth performance, serum metabolites and gene expression of heat‐stressed growing quails (Naz, Bibi et al. 2024). Specifically, the inclusion of 0.2 mg/kg of nano‐Se in the diets of these quails has been shown to enhance growth performance without negatively affecting carcass characteristics, blood parameters or gene expression (Naz, Bibi et al. 2024). Supplementation of BNSe at levels of 0.4 and 0.6 g/kg significantly enhanced growth performance, haematological indices and antioxidant capacity in Japanese quails (Reda et al. 2023). The addition of BNSe improved feed conversion rates and increased beneficial lactic acid bacteria while reducing harmful pathogens such as *E. coli* and *Salmonella*. Notably, BNSe also elevated AST activity and blood Se levels, demonstrating its positive effects on overall health and productivity in quails (Reda et al. 2023). In another study, the impact of nano copper in semi‐purified diets for laying quails and its effects on performance and metabolic state was investigated (Lopes et al. 2024). A total of 160 quails were used in a completely randomized design, incorporating different copper sources, including copper sulphate, CuO and NCuO at levels of 200, 400 and 800 ppm (Lopes et al. 2024). The results indicated that adding NCuO up to 800 ppm to the diets did not negatively affect productive performance and provided higher bioavailability compared to conventional CuO. Additionally, haemoglobin and haematocrit levels increased with the inclusion of 200 and 400 ppm of NCuO (Leite et al. 2024). A study aimed to evaluate the effects of low (6 mg/kg) and high (18 mg/kg) doses of CuO‐NPs on growth performance, DNA integrity and the histological structure of the liver, kidney and heart in 120 1‐day‐old Japanese quails divided into three groups (Naz, Raza et al. 2024). Results showed that the 6 mg/kg group had significantly higher feed intake and BWG compared to the 18 mg/kg group, which also exhibited greater DNA damage and histopathological changes. Thus, the findings indicate that although the lower dose may promote growth, the higher dose is harmful, leading to impaired growth and damage to vital organs (Naz, Raza et al. 2024). HS constitutes one of the most critical environmental challenges faced by poultry production. This condition is particularly concerning in quail farming, as it can result in decreased productivity, higher mortality rates and various health issues among quails. Nano‐selenium (nano‐Se) has emerged as a promising approach for mitigating HS in growing quails (Mahmoud et al. 2024). Its properties, which include antioxidant, immunomodulatory and cellular protective effects, can contribute to the maintenance of quail health and performance during elevated temperature conditions (Shirsat et al. 2016). Incorporating nano‐Se into the poultry diet has been demonstrated to significantly enhance feed efficiency, growth performance and immunological responses (Pelyhe and Mézes 2013; Marković et al. 2018).

Recent reports indicate that the supplementation of nano‐curcumin has positive effects on the growth, lipid profile, blood constituents, antioxidant indices and immunity of growing quail (Reda et al. 2020). It has been shown that the supplementation of chemical nano‐Se at levels of up to 0.4 g/kg can enhance performance, improve lipid profiles, boost antioxidant indices and strengthen immunity, while also reducing intestinal pathogens in quails during the fattening period (1–5 weeks of age) (Alagawany et al. 2021). It is advisable to supplement the diet with SeNPs to achieve a Se level of 0.5 mg/kg, as this has been shown to mitigate aflatoxin B1 toxicosis in broiler quails (Khazraei et al. 2021). A study investigated the impact of CNPs as a feed additive on various parameters in Japanese quail from Day 1 to 42 (El‐Ashram et al. 2020). A total of 240 quails were divided into four groups, with Group 4 receiving the highest dosage of 200 mg CNPs/kg diet, resulting in significantly better final weight and BWG (El‐Ashram et al. 2020). Although CNPs did not affect feed intake or mortality, it improved FCR in groups 2 and 4. Notably, CNPs groups showed healthier intestinal bacteria profiles and lower abdominal fat compared to controls, indicating a beneficial effect on the growth performance of the quail (El‐Ashram et al. 2020). Furthermore, a study investigated the use of cysteine‐coated Fe_3_O_4_ NPs as a replacement for FeSO_4_ in quail diets (Rahmatollah et al. 2017). The results indicated that the negative control group exhibited the lowest BWG, whereas quails receiving 1.2 mg/kg of cysteine‐coated iron NPs (Fe‐NPs) demonstrated comparable growth and FCR to those on the positive control with FeSO_4_ (Rahmatollah et al. 2017). These findings suggest that 1.2 mg/kg of cysteine‐coated Fe_3_O_4_ NPs is adequate for optimal growth and nutrition in quails, confirming their potential as an effective dietary iron source (Rahmatollah et al. 2017). A further study aimed to examine the influence of l‐cysteine‐coated iron oxide NPs on the reproductive performance of breeding quails (Mohammadi et al. 2017). The findings revealed that quails fed a diet supplemented with 6 mg/kg of Fe_3_O_4_‐Cys NPs showed significantly greater egg production and mass compared to control groups. Additionally, the group receiving 60 mg/kg of these NPs recorded the highest egg weights, whereas both iron supplements improved fertility and resulted in heavier day‐old chicks. The results suggest that l‐cysteine‐coated Fe_3_O_4_ NPs enhance iron availability and may serve as effective feed additives for quails (Mohammadi et al. 2017). In another study, the effect of varying quantities of ZnONPs on growth and mucosal enzyme activity in Japanese quails was examined (Ahmadi and Rahimi 2023). The results indicated that live weight was higher in the groups receiving 40 and 60 mg of ZnONPs compared to the control group, and the FCR improved (Ahmadi and Rahimi 2023). Additionally, amylase and lipase activity increased in the groups receiving the NPs, although these changes were not statistically significant (Ahmadi and Rahimi 2023). In a separate experiment, the effect of CrNPs supplementation on physiologically stressed Japanese quails was evaluated (Berenjian et al. 2017). The results indicated that increased levels of nano‐Cr improved live BW, enhanced energy and protein utilization efficiency and increased white blood cell count (Berenjian et al. 2017). Additionally, a positive correlation was observed between nano‐Cr levels and feed intake as well as ADG, whereas the negative effects of physiological stress were alleviated (Berenjian et al. 2017). Similarly, the dietary supplementation of BNSe at levels of 0.4 and 0.6 g/kg significantly enhanced growth performance, improved haematological parameters, elevated antioxidant capacity and increased immunological indices in Japanese quails (Reda et al. 2023).

### 7.5 Dietary Impacts of NPs in Horse

**FIGURE 6:**
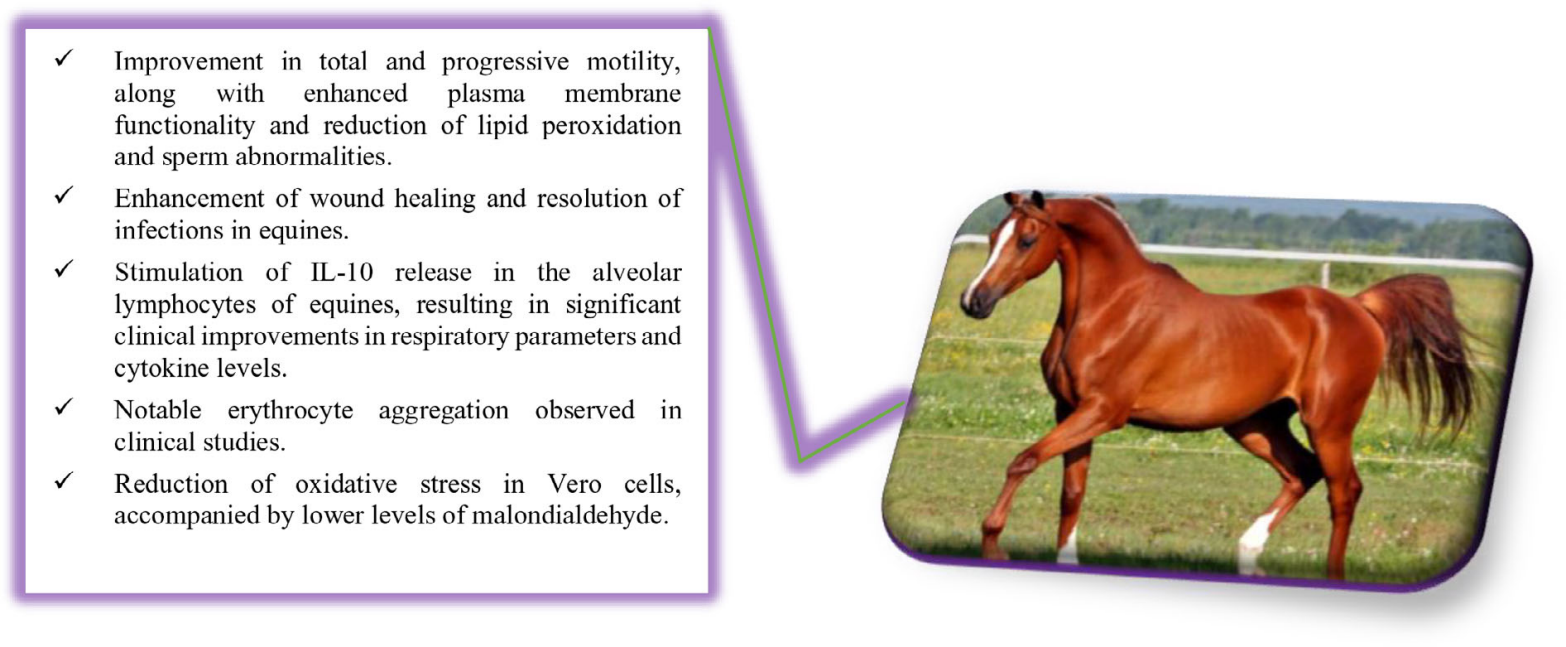
Main effects of nanoparticles in horse.

Horses have been an integral part of human life since ancient times, and following the industrial revolution, their breeding for recreational and competitive purposes began to gain prominence in developed countries. The nutritional requirements for horses raised for sport and leisure activities are influenced by several factors, including their physiological condition, age, sex and performance level, resulting in a feeding regimen that has become almost conventional (Evci 2024). Improperly formulated diets for horses can lead to significant health issues, causing metabolic disorders that adversely affect both the well‐being of the horses and the interests of their owners (Evci 2024). Despite the existence of numerous studies concerning the application of NPs in livestock species, much of this research is predominantly focused on ruminants and laboratory animals. Nevertheless, in equine nutrition, the incorporation of nanomaterials has primarily centred around preventive and therapeutic applications (Gopi et al. 2017; Xie et al. 2018; Elghandour et al. 2018; Adegbeye et al. 2019). An illustration of the main effects of NPs in horse is presented in Figure 6. As presented in the figure, NPs demonstrate significant benefits in equine health: They improve sperm quality (enhancing motility and membrane integrity while reducing abnormalities), accelerate wound healing and infection resolution and modulate immune responses by stimulating IL‐10 production in lung lymphocytes, leading to better respiratory function. Additionally, NPs reduce oxidative stress (lowering MDA levels in cells) and show notable effects on erythrocyte aggregation in clinical settings. The utilization of NPs as feed additives, especially in the context of immunotherapy, is justified by their favourable digestibility through the intestinal lumen (Hill and Li 2017; Hameed 2021). Se is a powerful antioxidant that plays a critical role in the synthesis of selenoproteins. Selenoenzymes, such as glutathione (GSH) peroxidases, along with seleno‐amino acids like l‐selenocysteine and l‐selenomethionine, serve as substitutes for sulphur in protein structures (Khan et al. 2024). Specifically, GSH peroxidase 4 is a crucial component that affects male fertility and the quality of sperm. Consequently, reduced levels of Se in selenoproteins can make spermatozoa more vulnerable to oxidative stress (Khan et al. 2024; Yuan et al. 2024). Oxidative damage to sperm during cooled storage poses a serious challenge, but SeNPs may offer a promising solution. In this context, a study evaluated the effects of SeNPs in the INRA96 extender on the quality of Turkmen stallion sperm over 72 h of cooled storage (Ghorbani et al. 2024). Twenty‐five ejaculates were treated with varying concentrations of SeNPs (0, 0.5, 1.0 and 1.5 µM) (Ghorbani et al. 2024). Results showed that although sperm quality declined significantly after 48 h, SeNPs improved total and progressive motility, plasma membrane functionality, and reduced lipid peroxidation and abnormalities, indicating their potential to enhance sperm preservation (Ghorbani et al. 2024). The manipulation of materials at the nanoscale offers a diverse array of properties that can be advantageous in equine nutrition. However, the integration of NPs into equine diets does not always produce clear and consistent results (Reddy et al. 2020). Additionally, there are currently no established limits regarding the concentrations of nano‐feed additives permissible for use in equines. Thus, supporting the use of any NP as a feed additive necessitates the availability of precise information concerning the potential toxicity of these specific compounds to the host (Reddy et al. 2020). Moreover, the relationship between the beneficial effects of various nano‐feed additives and their respective concentrations remains insufficiently understood, even in other livestock species. Consequently, proper characterization and toxicological assessments of nano‐feed additives are essential prerequisites for their safe application in equines (Reddy et al. 2020). Metallic and phyto‐based synthesized NPs, including Ag, gold, calcium, iron, Se, silicon, titanium and Zn, are utilized in research pertaining to animal nutrition and play a significant role in various domains of nanotechnology (Adegbeye et al. 2019). Recent advancements in the fields of nanoscience and nanotechnology have the potential to be applied to equine production systems and nutrition. These applications may include the incorporation of feed additives aimed at enhancing health and productivity, improving mineral bioavailability and aiding in the removal or inactivation of toxins present in animal feeds (Adegbeye et al. 2019). Research on the use of NPs in equine nutrition is limited; however, there are other studies regarding the application of NPs for horses, some of which will be discussed in the following sections.

A study evaluated the wound healing effects of green and chemical ZnONPs in equines, using 10 animals with either infected or non‐infected wounds (Metwally et al. 2020). Six animals presented visible signs of infection, whereas four had fresh wounds. Throughout 3 weeks of daily topical treatment, macroscopic wound contraction percentages for the green ZnONPs gel were 45, 76 and 93.6, compared to 40.4, 67.2 and 90.6 for the chemical gel. The results indicate that the green NPs gel significantly enhances wound healing and infection resolution in equines (Metwally et al. 2020). In a study, the use of GNPs as biodegradable carriers for delivering active ingredients to the lungs has shown potential for non‐invasive administration (Fuchs et al. 2012). The research explored the aerosolization of both plain and cytosine–phosphate–guanine oligodeoxynucleotides (CpG‐ODN)‐loaded GNPs using pressured metered dose inhalers and vibrating‐mesh nebulizers. Findings revealed that GNP sizes post‐nebulization were approximately 248.2 and 222.3 nm for active and passive nebulizers, respectively, with a consistent GNPs concentration and significant particle deposition (Fuchs et al. 2012). Notably, nebulized CpG‐ODN‐loaded GNPs effectively stimulated IL‐10 release in equine alveolar lymphocytes, establishing a foundation for GNP‐mediated pulmonary immunotherapy in vivo (Fuchs et al. 2012). Additionally, a study investigated the use of a nebulized GNP‐CpG formulation (CpG‐GNP), both alone and with specific allergens, for treating spontaneous allergic asthma in horses as a parallel to human asthma (Klier et al. 2017). Results indicated that administration of CpG‐GNP, regardless of allergen inclusion, led to significant clinical improvements in respiratory parameters and cytokine concentrations, suggesting its potential as an effective immunotherapy for allergic asthma in both equines and humans (Klier et al. 2017). In another study, the haemolytic effects and oxidative stress caused by ZnONPs and their polymeric hydrogels were examined on horse erythrocytes and Vero cells (Raguvaran et al. 2017). The findings indicated that higher concentrations of ZnONPs led to significant erythrocyte aggregation, whereas ZnONP‐loaded hydrogels mitigated oxidative stress in Vero cells, as shown by reduced MDA levels. Overall, the study highlights the protective role of polymeric hydrogels in reducing the toxicity of ZnONPs on mammalian cells (Raguvaran et al. 2017). In general, the evaluation of potential threats and risks associated with nano‐feed additives should be supported by the establishment of precise and clear criteria for their identification. Tissue retention of NPs serves as a preliminary indicator of toxicity. Therefore, any research focused on advocating for NPs as feed additives must assess the extent of their tissue retention. Until all the aforementioned limitations are thoroughly addressed, the application of nano‐feed additives in equines remains inappropriate (Reddy et al. 2020).

### 7.6 Impacts of NPs in Pig

**FIGURE 7:**
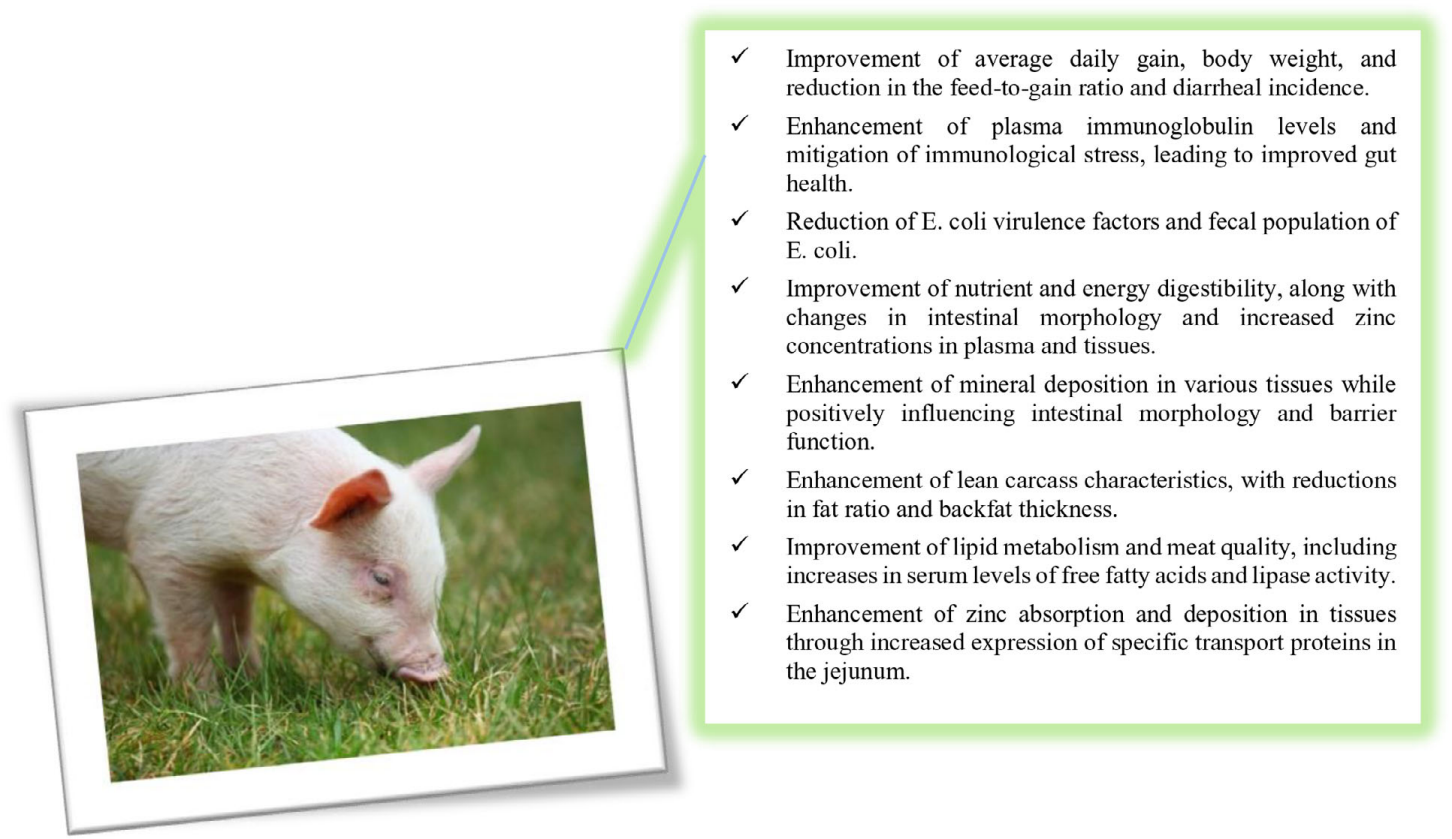
Main effects of nanoparticles in pig.

The integration of innovative nano additives into the animal feed supply chain necessitates specific considerations that contribute to a thorough assessment of exposure and hazard characterization of these nanomaterials. It is essential to consider in vitro and in vivo studies, along with toxicological research, to accurately predict the behaviour and fate of NPs. In this context, a study examined the inclusion of a novel nano‐Ag material in pig and chicken feed as a growth‐promoting additive, highlighting the need for a comprehensive risk assessment involving in vitro gastrointestinal digestions to assess its degradation and NP exposure (Ben‐Jeddou et al. 2024). The research utilized inductively coupled plasma mass spectroscopy to investigate the release of Ag across different digestion phases. Findings revealed that 8%–13% of the total Ag was bioaccessible during the intestinal phase, predominantly in the form of dissolved Ag species, whereas less than 0.1% was present as Ag‐containing particles. Furthermore, the additive exhibited varying behaviour in different species, with the feed matrix playing a crucial role in determining the fate of Ag (Ben‐Jeddou et al. 2024). In a separate study, the influence of CNPs supplementation on growth, immune response and gut microbiota in weaned pigs was assessed (Xu et al. 2019). The findings indicated that higher concentrations of CNPs positively impacted ADG and reduced feed‐to‐gain ratio and diarrhoeal incidence, with no significant change in feed intake (Xu et al. 2019). Additionally, CNPs enhanced plasma immunoglobulin levels and affected cytokine responses post‐lipopolysaccharide challenge, suggesting its potential to mitigate immunological stress and improve gut health. These results imply that CNPs could serve as a beneficial functional feed additive in piglet diets (Xu et al. 2019). Zn is a vital trace element for animals, significantly contributing to their nutrition, growth and immune function. Due to its effectiveness and cost‐effectiveness, Zn in the form of ZnO has traditionally been administered in high doses (ranging from 2000 to 3000 mg/kg of diet) to weaned piglets as a substitute for antibiotics, aimed at preventing intestinal inflammation and promoting BWG (Hu et al. 2012; Kociova et al. 2020). However, starting in 2022, the European Union has implemented a ban on the use of Zn at such elevated levels (Kociova et al. 2020). ZnNPs present a promising alternative to large doses of Zn, offering enhanced pharmacokinetic efficiency, particularly when addressing coliform bacterial infections in various mammals (Horky et al. 2019). In this regard, the study compared two Zn phosphate‐based NP formulations (ZnA and ZnC) in weaned piglets (Kociova et al. 2020). The findings revealed that piglets supplemented with ZnA exhibited significantly greater BWG compared to the control group, whereas the prevalence of *E. coli* virulence factors was notably reduced in those receiving ZnC (Kociova et al. 2020). Overall, Zn phosphate NPs show promise as effective replacements for ZnO in enhancing piglet growth performance and potentially reducing environmental contamination (Kociova et al. 2020). Biogas, a biofuel derived from anaerobic digestion, serves various applications, including thermal energy production and power generation. Primarily composed of 50%–70% methane and 30%–50% CO_2_, it can be refined to meet natural gas standards for transport and grid injection (Cerrillo et al. 2021). Current upgrading techniques include energy‐intensive methods like scrubbing and pressure swing adsorption; however, emerging technologies such as electromethanogenesis show promise in converting CO_2_ into methane. Ongoing research explores strategies like bioaugmentation, co‐digestion and the utilization of NPs to enhance methane production in anaerobic digestion (Cerrillo et al. 2021). In this regard, a study investigated the impact of zero‐valent iron NPs (nZVI) on methane production during the anaerobic digestion of pig slurry (Cerrillo et al. 2021). The findings revealed that nZVI inhibited methane production in mesophilic conditions across all tested dosages, whereas a lower dosage significantly enhanced methane production in thermophilic settings (Cerrillo et al. 2021). In continuous operations, the addition of nZVI resulted in methane content reaching 80%–85%, with average production rates increasing by 165% in thermophilic conditions and 94% in mesophilic conditions compared to controls. These results suggest that incorporating nZVI could effectively enhance in situ biogas upgrading during anaerobic digestion (Cerrillo et al. 2021). Post‐weaning diarrhoea (PWD) represents one of the most prevalent diseases in pig farming, resulting in significant economic losses. Effective management of PWD involves various strategies, including immunization, the use of feed additives, breeding pigs with resistance, probiotics and bacteriophages (Drider et al. 2022). Furthermore, materials based on different types of NPs can be utilized with the goal of developing innovative methods for drug delivery, rationalizing antibiotic use and addressing key challenges associated with infections in animal health (Drider et al. 2022). An illustration of the main effects of NPs in pig is presented in Figure 7. As shown in the figure, NPs enhance swine growth (weight gain, feed efficiency), gut health (immunity, pathogen control) and nutrient absorption while improving carcass quality (lean meat, reduced fat) and metabolism.

In this regard, a study was conducted to assess the potential of both free colistin and colistin‐loaded alginate NPs (colistin/AlgNPs) in selecting for colistin‐resistant enterotoxigenic *E. coli* in a controlled experimental setting (Drider et al. 2022). The findings indicated that colistin/AlgNPs exhibited a marginally superior therapeutic effect compared to free colistin (Drider et al. 2022). Although both treatment methods resulted in a temporary reduction in the overall faecal population of *E. coli* predominantly consisting of colistin‐resistant isolates, most of the dominant *E. coli* population were found to be susceptible at the conclusion of the trial. Further research is warranted to investigate the therapeutic effectiveness of colistin/AlgNPs under various experimental and field conditions (Drider et al. 2022). Weaning is crucial for pigs due to their physiological and immunological immaturity, which heightens their vulnerability to infections, particularly diarrhoea, and adversely affects growth (Lallès et al. 2007; Campbell et al. 2013). Consequently, the incorporation of feed additives has become a prevalent practice to modulate the immune system and intestinal microbiota, thereby enhancing the health and performance of pigs in the immediate post‐weaning phase (Lallès et al. 2007). In this regard, a study was conducted to assess the impact of varying dietary levels of ZnONPs on the growth and health of weanling pigs (Milani et al. 2017). The results indicated that although ZnONPs levels did not significantly enhance overall growth performance, a linear improvement in gain‐to‐feed ratio and a reduction in diarrhoea were noted during the initial days post‐weaning (Milani et al. 2017). Additionally, increasing ZnONPs levels correlated with improved nutrient and energy digestibility yet did not affect faecal Zn excretion, blood parameters or bacterial counts in the intestines (Milani et al. 2017). Overall, neither low ZnONPs levels nor a pharmacological dose of conventional ZnO were effective in enhancing the growth of weanling pigs, although some benefits in managing PWD were observed (Milani et al. 2017). In another study, the efficacy of ZnONPs was assessed as an alternative to colistin sulphate (CS) and ZnO in improving growth performance and intestinal health in weaned piglets (Wang et al. 2017). The findings revealed that a 1200 mg/kg supplementation of ZnONPs significantly enhanced BW and DWG compared to the CS group. Furthermore, ZnONPs positively influenced intestinal morphology and increased Zn concentrations in plasma and tissues, while exhibiting comparable effects on serum enzymes and intestinal health indicators to those of the combined CS and ZnO treatment (Wang et al. 2017). Nonetheless, a reduction in Zn levels was observed in the liver, tibia and faeces compared to the CS + ZnO group, suggesting potential as a viable substitute in piglet diets (Wang et al. 2017). In addition, another study demonstrated that dietary supplementation with 800 mg/kg of ZnONPs and 3000 mg/kg ZnO significantly improved ADG and reduced diarrhoea rates in weaned piglets compared to the control group (Wang et al. 2017). Furthermore, this supplementation enhanced mineral deposition in various tissues and positively influenced intestinal morphology and barrier function, suggesting that ZnONPs could serve as a viable alternative to higher levels of traditional ZnO in piglet diets (Wang et al. 2018). In addition, another study investigated the impact of chromium‐loaded CNPs (Cr‐CNPs) on various parameters in finishing pigs, revealing notable benefits (Wang et al. 2014). The findings indicated that the inclusion of Cr‐CNPs in the diet significantly enhanced the gain‐to‐feed ratio and lean carcass characteristics, while reducing both fat ratio and backfat thickness (Wang et al. 2014). Furthermore, the supplementation led to increased serum levels of free fatty acids and lipase activity, alongside a decrease in serum insulin levels, suggesting improved lipid metabolism. Overall, the results highlight the positive effects of Cr‐CNPs on growth performance and meat quality in pigs (Wang et al. 2014). In a study aimed at evaluating the effectiveness of sepiolite‐Ag and kaolinite‐Ag clay nanocomposites as carriers for AgNPs for oral administration in animal feed, the digestibility and release of Ag were assessed (Abad‐Álvaro et al. 2019). The results indicated that less than 1% of Ag was released during the stomach simulation, with higher release rates observed in the intestinal simulation, where kaolinite‐Ag showed a greater release than sepiolite‐Ag (17% vs. 7%). In vivo assessments in weaned pigs indicated that those administered kaolinite‐Ag retained more Ag in the liver, whereas sepiolite‐Ag yielded higher Ag levels in faeces. However, Ag concentrations in muscle tissues remained below detectable limits in all cases (Abad‐Álvaro et al. 2019). In another study aimed at examining the prolonged effects of dietary ZnONPs on organ weight, liver function and trace mineral dynamics in intrauterine growth retardation pigs, it was found that incorporating ZnONPs did not significantly alter organ development, liver function markers or trace mineral concentrations among the different piglet groups (Zhou et al. 2022). However, it notably enhanced Zn absorption and deposition in tissues by increasing the expression of specific transport proteins in the jejunum, without evidence of intact NPs in the gastrointestinal tract or organs (Zhou et al. 2022).

### 7.7 Dietary Impacts of NPs in Rat

**FIGURE 8:**
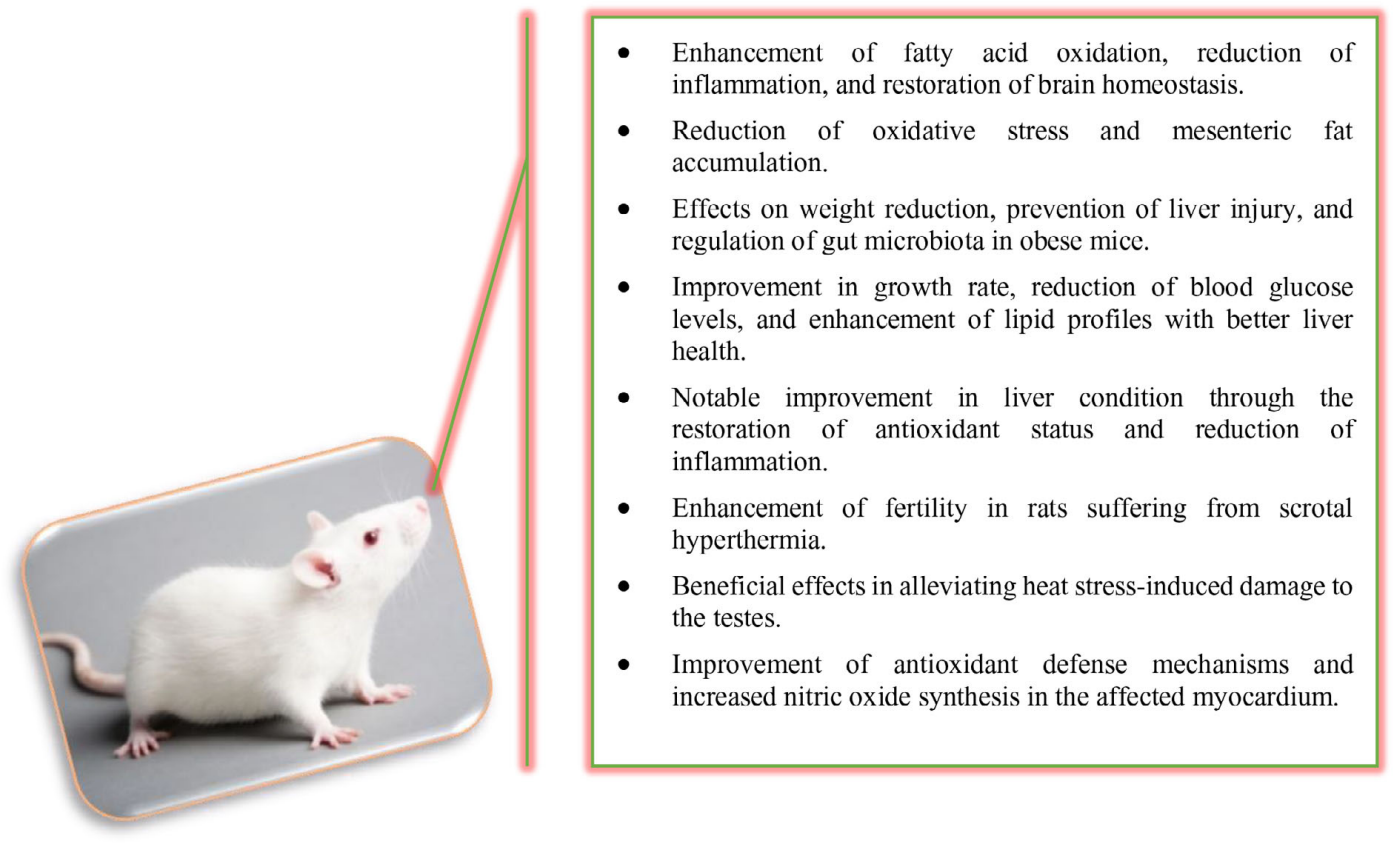
Main effects of nanoparticles in rat.

An illustration of the main effects of NPs in rat is presented in Figure 8. As shown in the figure, NPs demonstrate significant benefits in mice/rats: They enhance metabolic health by reducing obesity (fat oxidation, mesenteric fat), improving liver function (antioxidant restoration, inflammation reduction) and regulating blood glucose/lipids. NPs protect reproductive health (fertility improvement, testicular heat‐stress relief) and cardiovascular function (myocardial antioxidant defence, nitric oxide synthesis), while modulating gut microbiota and brain homeostasis. Obesity leads to inflammation in adipose tissue and can impact the central nervous system, resulting in oxidative stress and mitochondrial dysfunction. Consequently, it is essential to explore new therapeutic alternatives. Gold NPs may facilitate the delivery of carnitine to adipose tissue, thereby enhancing fatty acid oxidation, reducing inflammation and ultimately restoring brain homeostasis. In this regard, da Silva et al. (2024) investigated the impact of gold NPs combined with carnitine on neurochemical changes in obese mice. Male Swiss mice were fed either a normal or high‐fat diet for 10 weeks, with subsequent treatments of saline, gold NPs, carnitine or their combination starting in Week 6. The findings revealed that obesity induced oxidative damage and mitochondrial dysfunction; however, treatments with gold NPs and carnitine, both alone and in combination, showed promise in reducing oxidative stress and mesenteric fat accumulation (da Silva et al. 2024). The main challenge in utilizing anthocyanins (ACNs) in nutrition is their digestive instability. In the study of Cheng et al. (2024), blueberry ACNs‐loaded NPs were developed using GA as the carrier and soy lecithin‐derived liposomal vesicles as the targeting scaffold. Results indicated that the liposomal vesicles‐GA structure enhanced the stability and antioxidant activity of the NPs. Additionally, in a high‐fat diet‐induced obese mouse model, the NPs demonstrated superior effects on weight reduction, liver injury prevention and gut microbiota regulation compared to free ACNs. These findings present a promising strategy for the delivery of ACNs aimed at preventing fatty liver (Cheng et al. 2024).

In another obesity study, the effects of fucoxanthin (FX) and FX‐based NPs (FZ) on glucose and lipid metabolism in obese mice were investigated (Zhang, Dong et al. 2024). Interventions with FX, FZ and zein hydrolysate (ZH) resulted in improved growth rates, lowered blood glucose, enhanced lipid profiles and better liver health. They also demonstrated significant modulation of genes related to glucose metabolism and changes in gut microbiota, revealing distinct in vivo behaviours of FX and FZ linked to their digestion rates (Zhang, Dong et al. 2024). Overall, their findings underscore the potential of FX‐encapsulated treatments in influencing metabolic pathways and the important role of gut microbiota in addressing obesity‐related disorders (Zhang, Dong et al. 2024). Cerium oxide NPs (CeO_2_NPs, NM‐212) are recognized for their catalytic and antioxidant properties, finding uses in various industries, drug delivery and cosmetics (Han et al. 2024). It is commonly utilized in various applications, including catalysts for purifying automobile exhaust, polishing semiconductor insulation layers and display glass, as well as in cosmetics and solid fuel cells (Han et al. 2024). In this context, a study was conducted where CeO_2_NPs were administered orally to Sprague Dawley rats over a period of 13 weeks at doses of 0, 10, 100 and 1000 mg/kg BW per day, followed by a 4‐week recovery. Although haematological and clinical chemistry parameters exhibited some changes, particularly in the highest dose group, there were no significant adverse effects or abnormalities found in pathological evaluations. Consequently, a dose of 1000 mg is suggested as the ‘no observed adverse effect level’ for CeO_2_NPs in both male and female rats (Han et al. 2024). Similarly, the toxicity and organ distribution of CeO_2_NPs were assessed through both intravenous and oral administration in rats (Park et al. 2018). Following treatment with doses of 30 and 300 mg/kg, rats were euthanized 24 h later, and cerium concentrations were analysed in various tissues and excretions. Results showed that oral administration resulted in low cerium levels in blood and tissues, with most detected in faeces, indicating minimal gastrointestinal absorption. In contrast, intravenous injection led to high cerium concentrations, primarily in the liver and spleen, with significant toxicity observed in these cases, whereas no adverse effects were noted in the oral treatment groups (Park et al. 2018). The translocation rate of ingested NPs and the influence of food matrices on uptake are critical for health risk assessments. A study involved female Sprague Dawley rats (*N* = 4/group) receiving daily doses of cerium oxide (CeO_2_ NM‐212) NPs via gavage or chocolate spread for 1 or 2 weeks, followed by a 2‐week recovery (Berthing et al. 2022). Results indicated a dose‐ and time‐dependent accumulation of NPs in the liver and spleen, with concentrations ranging from 0.1 to 0.3 and 0.004 to 0.005 ppm, respectively. Although no significant difference in cerium levels was found between the two administration methods, microscopy indicated necrotic changes in the liver and reduced cellularity in the spleen. The use of snacks for NPs delivery offers a more humane and relevant exposure method compared to gavage (Berthing et al. 2022). Cobalt ferrite NPs (CFNs) are widely utilized in various fields, raising concerns about their potential health impacts due to increased exposure. Hassanen et al. (2023) investigated the pulmonary toxicity of CFN in rats, focusing on different dosage levels (0.05, 0.5 and 5 mg/kg BW). Their results indicated that higher doses of CFN lead to dose‐dependent oxidative stress, marked by elevated MDA levels and reduced GSH content, along with significant lung inflammation and damage. Notably, the lowest dose (0.05 mg) showed no significant toxicity, suggesting that daily oral intake of 0.5 or 5 mg CFN poses a risk of pulmonary toxicity through oxidative and inflammatory mechanisms. Their findings contribute to understanding the toxicological effects of CFN and inform risk assessment standards (Hassanen et al. 2023). Curcumin or curcumin CNPs have beneficial anti‐inflammatory, antioxidant and cytoprotective properties, but its low absorption limits its therapeutic application (Radwan et al. 2023). Researchers investigated the use of NPs to improve the bioavailability of curcumin in rats suffering from liver fibrosis induced by thioacetamide (TAA) (Radwan et al. 2023). Sixty rats were divided into six groups, and TAA exposure resulted in significant liver damage, marked by elevated liver enzymes, reduced albumin and protein levels, increased MDA, decreased antioxidant enzyme activity and higher inflammation markers (TNFα and DNA damage). Treatment with curcumin or curcumin CNPs notably improved liver condition by restoring antioxidant status and reducing inflammation. Notably, curcumin CNPs were more effective than standard curcumin, indicating that NP formulation significantly enhances curcumin's efficacy against TAA‐induced hepatotoxicity (Radwan et al. 2023). The findings of the study showed that exposure to iron superoxide NPs led to a decrease in sperm parameters, an increase in the expression of the pro‐apoptotic gene Bax, and a decrease in the expression of the anti‐apoptotic gene Bcl‐2 in rats (Deldar Abad Paskeh et al. 2024). These results indicate a negative impact of NPs on male fertility through increased apoptosis and reduced sperm quality. Additionally, the association between infertility and testicular hyperthermia is becoming increasingly evident, and the administration of iron superoxide NPs can have significant effects on male infertility (Deldar Abad Paskeh et al. 2024). It is also recommended to consider green synthesis of NPs in this field (Deldar Abad Paskeh et al. 2024). Research has indicated that iron particles loaded with curcumin may serve as an alternative treatment to enhance the spermatogenesis process in mice affected by scrotal hyperthermia (Afshar et al. 2020). Similarly, the use of curcumin NPs in a testicular hyperthermia model exhibited beneficial effects in alleviating damage to the testes caused by HS. This approach may serve as an effective strategy to address the challenges that impede the application of curcumin in conditions characterized by elevated intratesticular temperature (Khosravi et al. 2020). ZnONPs are widely utilized in cosmetics and are increasingly being assessed for drug delivery and tissue engineering. Recent findings indicate their potential cardiotoxicity and systemic toxicity (Hussein et al. 2024). Furthermore, Hussein et al. (2024) investigated the cardiac effects of ZnONPs (39 nm) on Wistar rats, using doses of 25, 50 and 100 mg/kg BW, assessing electrocardiogram (ECG) parameters, biochemical indicators and oxidative stress biomarkers (Hussein et al. 2024). Their results revealed a dose‐dependent impact, with the 100 mg/kg BW group exhibiting significant ECG alterations, increased heart enzymes (creatine kinase‐MB and lactate dehydrogenase), elevated oxidative stress markers (MDA and nitric oxide) and reduced GSH levels, accompanied by histopathological damage to cardiac tissues. Conversely, the bulk ZnO group at the same dose did not affect heart function significantly. Their findings suggest that ZnONPs can induce cardiac dysfunction and lesions at higher doses (Hussein et al. 2024). Conversely, it has been reported that ZnONPs can cause significant damage to cardiomyocytes, characterized by reduced cell proliferation, increased oxidative stress, cell death and pronounced morphological alterations that disrupt cellular integrity (Mendoza‐Milla et al. 2022). Consequently, the consumption of products containing ZnONPs may lead to the development of organ failure and cardiovascular diseases, thereby posing a potential threat to heart health (Mendoza‐Milla et al. 2022). The study demonstrated that ZnONPs significantly improved the antioxidant defence mechanisms and increased nitric oxide synthesis in the affected myocardium, suggesting their potential as therapeutic agents for mitigating doxorubicin‐induced cardiotoxicity (Mohamed et al. 2024). Research indicates that chronic consumption of a high‐fat diet can adversely affect bone metabolism, but this negative impact may be alleviated through the supplementation of CrNPs (Cholewińska et al. 2024). In contrast, altering dietary habits alone does not produce similar protective effects on bone health (Cholewińska et al. 2024). Toxicological concerns regarding NPs consumption primarily arise from excessive intake at high doses. For instance, a rat study demonstrated dose‐dependent hepatotoxicity of ZnONPs, with ≥10 mg/kg doses inducing liver damage via oxidative stress, NLRP3 inflammasome activation and apoptosis (verified by TUNEL assay), whereas 5 mg/kg exerted protective effects (Mirzaei et al. 2025). Histopathological alterations and biochemical markers established this dose‐dependent toxicity threshold, illustrating NPs’ dual nature as either therapeutic agents or potential toxins depending on concentration.

### 7.8 Dietary Impacts of NPs in Rabbit

**FIGURE 9:**
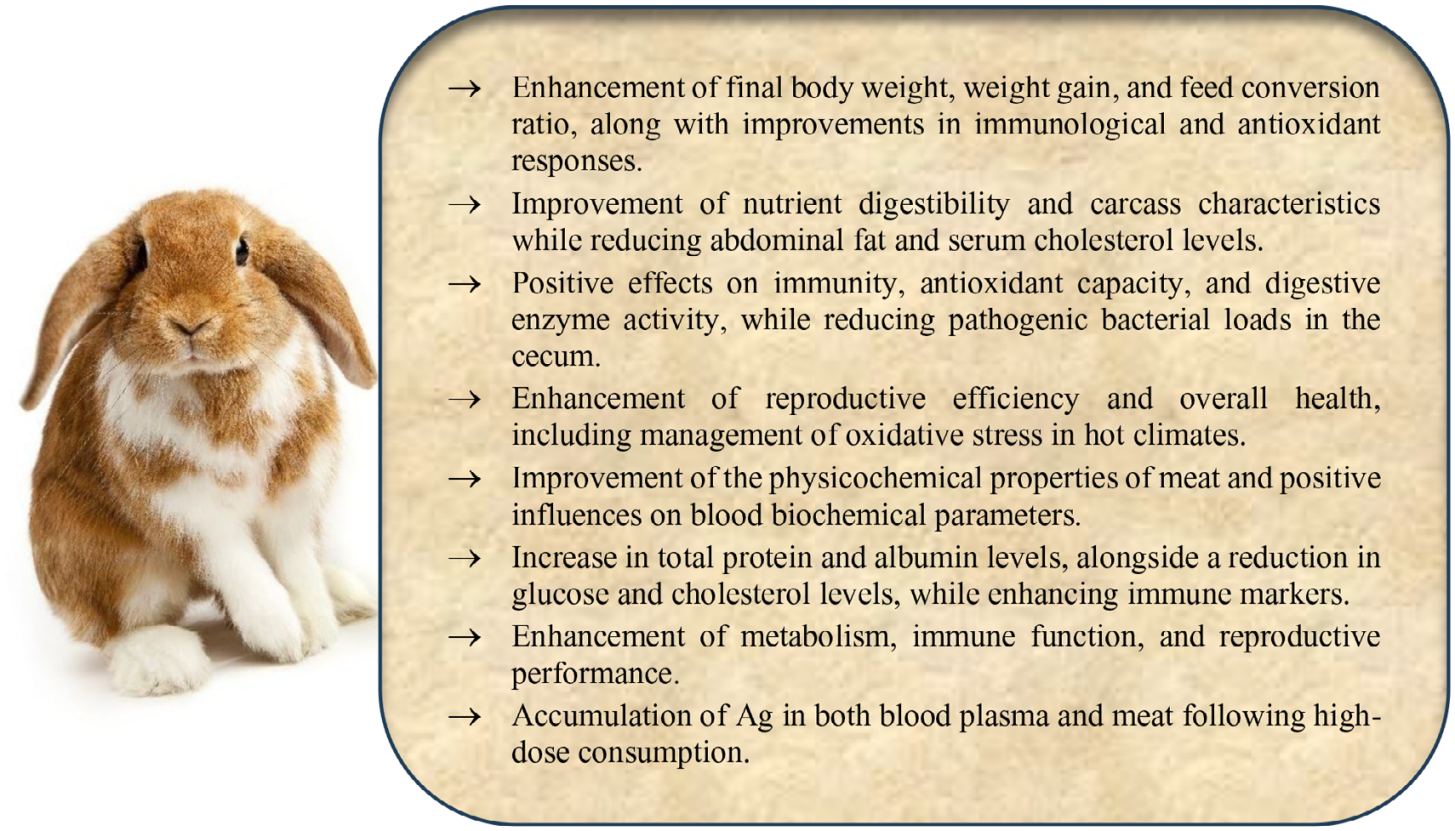
Main effects of nanoparticles in rabbit.

An illustration of the main effects of NPs in rabbit is presented in Figure 9. As explained in the figure, NPs enhance mice growth, immunity and metabolism while improving gut health and meat quality, though high doses may cause Ag accumulation. HS significantly affects rabbit growth, health and productivity. A study investigates the effects of dietary mineral NPs (Se and Zn) and *Spirulina platensis* (SP) on heat‐stressed growing rabbits (Bashar et al. 2023). A total of 180 weaned New Zealand White rabbits were divided into six treatment groups over an 8‐week summer period, receiving either a control diet or diets supplemented with SP, SeNPs, ZnNPs or combinations thereof. The results showed that dietary supplementation significantly enhanced final BW, BWG, FCR and blood parameters and improved immunological and antioxidant responses (Bashar et al. 2023). The findings suggest that incorporating SP and SeNPs can effectively mitigate the adverse effects of HS, promoting better health and heat tolerance in growing rabbits (Bashar et al. 2023).

Similarly, the incorporation of *Spirulina* alongside SeNPs has a synergistic impact, making it an effective dietary supplement for enhancing reproductive efficiency, overall health, oxidative stress management and immune function in rabbits’ bucks, particularly in breeding strategies implemented in hot climates (El‐Ratel et al. 2023). According to the research conducted by Sheiha et al. (2020), the administration of biologically synthesized nano‐Se at dietary levels of 25 or 50 mg/kg significantly enhanced the growth performance, renal and hepatic function, carcass characteristics, antioxidant status and inflammatory cytokine profiles in growing rabbits subjected to thermal stress. Abdel‐Wareth et al. (2024) examined the effects of ZnONPs and SeNPs on the growth performance and health of rabbits. A total of 80 New Zealand White rabbits, averaging 30 days old and weighing 720 ± 6 g, were assigned to four dietary treatments over 60 days: a control diet, a control diet supplemented with 40 mg/kg ZnONPs, a control diet with 0.6 mg/kg SeNPs and a combined diet of both supplements. Their findings indicated that supplementation significantly enhanced growth performance, carcass yield and digestibility of nutrients, while also reducing abdominal fat and serum cholesterol levels. However, liver concentrations of Zn and Se remained unaffected by the dietary treatments (Abdel‐Wareth et al. 2024). Similarly, Abdel‐Wareth et al. (2022) proposed that the addition of ZnONPs at doses ranging from 20 to 80 mg/kg can alleviate the adverse effects of HS on the performance and health of rabbits. This supplementation was found to enhance growth performance, improve the physicochemical properties of meat and positively influence the blood biochemical parameters in White New Zealand rabbits (Abdel‐Wareth et al. 2022). In a study, the influence of zinc oxide‐CNPs (Zn‐CNPs) on various parameters in rabbit diets, including growth performance, plasma constituents, carcass indices and immune function, was investigated (Hassan et al. 2023). Eighty male V‐line rabbits, aged 5 weeks, were assigned to one of four dietary groups: control, 50, 75 and 100 ppm Zn‐CNPs. The results showed that Zn‐CNPs did not affect final BW or ADG; however, 100 ppm Zn‐CNPs significantly improved nutrient digestibility and carcass weights (Hassan et al. 2023). Additionally, plasma analyses indicated increased levels of total protein and albumin for the 75 and 100 ppm groups, alongside reduced glucose and cholesterol levels while enhancing immune markers. Ultimately, Zn‐CNPs can be safely incorporated into rabbit diets to boost health and immune functions without negatively impacting growth performance (Hassan et al. 2023). A study demonstrated that dietary supplementation of AgNPs at concentrations of 0.25–1.00 mg/kg significantly enhanced live BW, FCR and BWG in growing New Zealand White rabbits (Kotb et al. 2024). Additionally, serum protein levels increased, whereas liver and kidney function indicators improved, with particular benefits observed at the 0.25 and 0.75 mg/kg levels. Furthermore, Ag‐NP supplementation positively affected immunity, antioxidant capacity and digestive enzyme activity, while reducing pathogenic bacterial loads in the cecum, highlighting its beneficial effects on overall health and performance (Kotb et al. 2024). In contrast, the administration of AgNPs to rabbits did not significantly impact ADG or FCR compared to control group, nor did it adversely affect carcass quality, blood biochemical parameters or antioxidant profiles. Notably, increased doses of AgNPs resulted in higher levels of Ag accumulation in both blood plasma and meat, raising health concerns for consumers (Abdelsalam et al. 2019). The study involved 48 weaned male New Zealand White rabbits, aged 6 weeks and weighing an average of 709.67 ± 13 g, which were randomly assigned to six groups to assess the impact of different Se forms (inorganic, NPs and organic) on their performance (K‐EMEl et al. 2024). The control group received a standard diet, whereas the experimental groups were given diets supplemented with varying levels of nano‐Se and organic Se from salinized yeast. Results showed that rabbits receiving nano‐Se exhibited superior digestibility, live BW, DWG, hot carcass weight and dressing percentage. Furthermore, both FCR and economic efficiency were enhanced in the nano‐Se groups compared to control, indicating that nano‐Se is more effective than inorganic Se for improving rabbit performance and economic viability (K‐EMEl et al. 2024). Furthermore, the supplementation of both free and nano‐encapsulated *Moringa oleifera* leaf ethanolic extract resulted in enhanced metabolism, immune function, milk yield and reproductive performance across various reproductive stages (El‐Desoky et al. 2022). Notably, the nano‐encapsulated form enabled an 80% reduction in the optimal dosage from 50 mg/kg BW to just 10 mg/kg BW, while maintaining treatment efficacy. This underscores the critical role of nano‐encapsulation in enhancing the bioavailability of fatty acids (El‐Desoky et al. 2022). Research indicates that both dietary CuNPs and copper acetate are more effective than CuO in promoting growth, suggesting their potential as beneficial additives in the nutrition of fattening rabbits (Al‐Sagheer et al. 2022). A study found that incorporating 7% nanopupunha (peach palm heart sheaths) into rabbit diets did not significantly impact overall performance but did enhance intestinal health and elevated the count of lactic acid bacteria in the cecum of growing rabbits (de Oliveira et al. 2023). Excessive use of NPs may cause toxicity in animals. For example, a study demonstrated dose‐dependent nephrotoxicity from CuO‐NPs in rabbits, showing elevated kidney biomarkers (blood urea nitrogen, uric acid, creatinine) and tissue damage (hyperaemia, inflammation) after 15‐day exposure, highlighting potential toxicity concerns in this regard (Yegeubayeva et al. 2024).

### 7.9 Dietary Impacts of NPs in Fish

**FIGURE 10:**
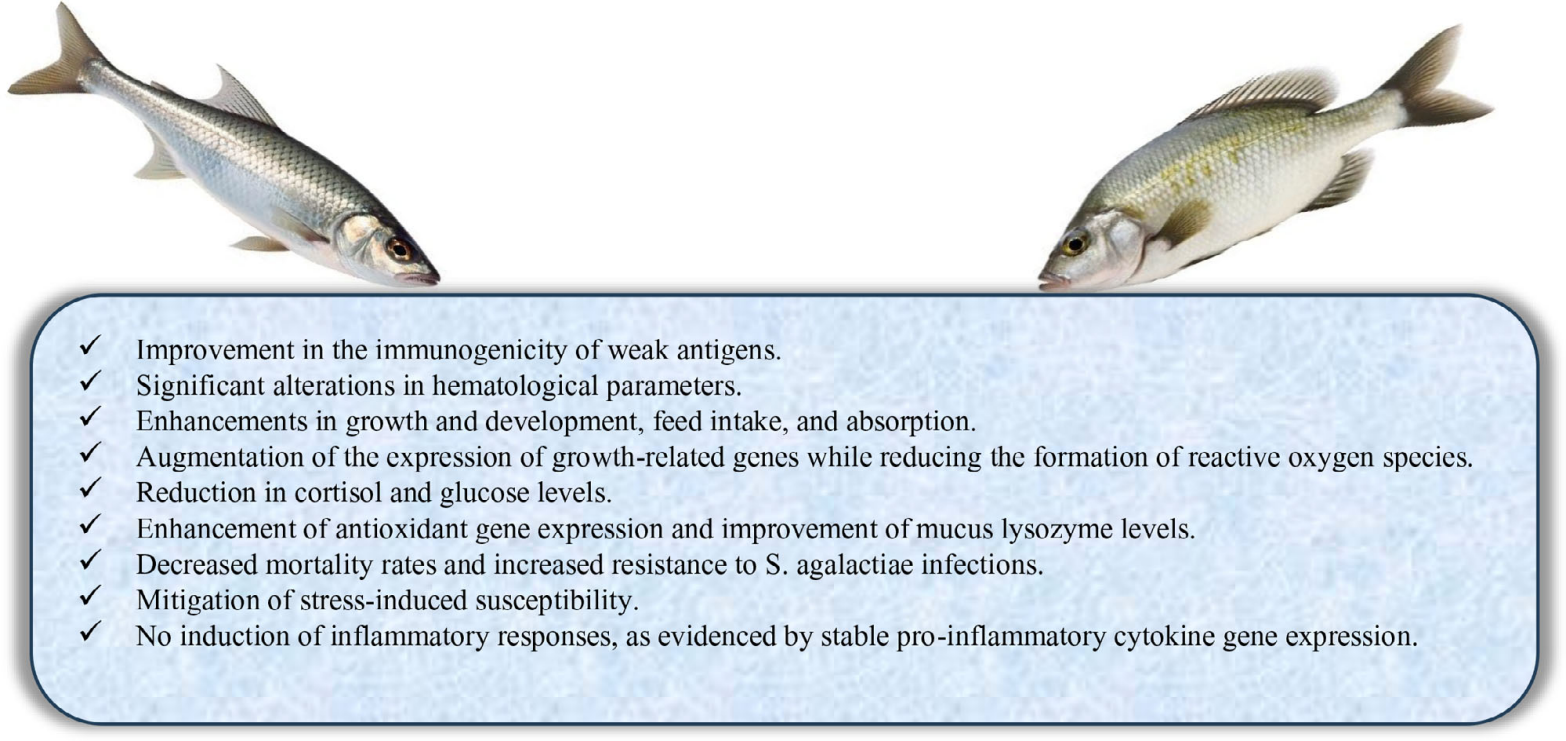
Main effects of nanoparticles in fish.

An illustration of the main effects of NPs in fish is presented in Figure 10. As explained in the figure, NPs enhance fish health and productivity by boosting immunity (improved antigen response, disease resistance against *Streptococcus agalactiae*), growth performance (feed intake, growth‐related genes) and stress resilience (reduced cortisol, oxidative stress). They improve antioxidant capacity (gene expression, lysozyme levels) and haematological parameters without triggering inflammation (stable cytokines), while lowering mortality rates. There is a continuous demand for the advancement of effective vaccines and delivery systems to prevent and manage both emerging and re‐emerging infectious diseases in aquaculture (Shirsat et al. 2016). Numerous infectious diseases present significant challenges in the development of effective vaccines. This difficulty primarily arises from the inability to create vaccines that elicit appropriate immune responses. The introduction of NPs has opened up substantial opportunities for designing vaccine delivery systems that efficiently target delivery, enhance the stability of antigens and function as effective adjuvants (Shirsat et al. 2016). Many NPs can penetrate antigen‐presenting cells through various pathways and promote suitable immune responses to the antigens (Shirsat et al. 2016). A variety of NPs are employed in fish vaccine delivery, including biodegradable polymers, nanoliposomes, carbon nanotubes, calcium phosphate and immunostimulating complexes. Among these, poly(lactic‐co‐glycolic acid) and chitosan have been the most extensively researched forms of NPs to date. Therefore, further exploration of additional forms of NPs is warranted (Shirsat et al. 2016). NPs have been found to improve the immunogenicity of weak antigens, offering several advantages compared to traditional adjuvant methods in aquaculture (Shirsat et al. 2016). These advantages include superior release kinetics, enhanced stability and the ability for targeted delivery (Shirsat et al. 2016). The presence of NPs in aquatic ecosystems poses significant threats to the organisms residing within these environments (Kahlon et al. 2018). These NPs, characterized by their diminutive size and unique physicochemical properties such as a high surface area, various shapes and the tendency to aggregate, are key contributors to the detrimental effects observed in aquatic habitats (Bakand et al. 2012). Estimates suggest that global production of ZnONPs ranges from 0.1 to 1.2 million tons (Srivastava et al. 2020; Janani et al. 2021). Given their extensive applications, ZnONPs are frequently introduced into aquatic ecosystems via wastewater discharge (Scown et al. 2010). The concentration of ZnONPs in surface waters is estimated to be approximately 0.001–0.058 µg/L, whereas in soil, it is around 0.024–0.661 µg/kg, and in sewage treatment effluent, it ranges from 0.22 to 1.42 µg/L (Gottschalk et al. 2009). ZnONPs adversely affect fish health by disrupting their physiological and biochemical functions (Shirsat et al. 2016). A study on *Oreochromis mossambicus* exposed them to 20 ppb of ZnONPs and related substances for 96 h, revealing significant changes in haematological parameters (Khan et al. 2022). Notable increases in white blood cells and platelets suggested an enhanced immune response, whereas decreases in red blood cells, haemoglobin and haematocrit levels were observed, along with structural damage to gill tissues, including disorganization and congestion (Khan et al. 2022). Plastics comprise a diverse array of polymers, including residual monomers and additives, making them affordable, lightweight and durable materials utilized extensively across various industries such as packaging, construction and textiles (Zaman et al. 2024). Micro‐ and nano‐sized plastics pose significant hazards as they can be ingested by various aquatic organisms, leading to bioaccumulation and toxicity, which impacts higher trophic levels in the food web (Cole et al. 2013; Martyniuk et al. 2023; Gholamhosseini et al. 2023; Pastorino et al. 2023). Additionally, studies have shown that these particles can cause adverse effects on fish, including immunotoxicity, digestive dysfunction and genotoxicity, raising concerns about their implications for aquatic biodiversity and human health (Schirinzi et al. 2020; Aliko et al. 2022; Zaman et al. 2024). Ingestion of particulate plastics, such as polystyrene NPs (PS‐NPs), can lead to significant physiological, biochemical and histological impairments in aquatic organisms, including gut blockage and immunological dysfunction (Zaman et al. 2024). Hence, a study aimed to investigate the effects of waterborne PS‐NPs on the physiological and immunological responses of the fish species *Hypophthalmichthys molitrix*. Over a period of 15 days, fingerlings were exposed to various concentrations of PS‐NPs (0.5, 1.0 and 2.0 mg/L) (Zaman et al. 2024). The findings revealed that higher concentrations of PS‐NPs led to significant adverse effects, including alterations in blood parameters and metabolic enzymes, increased stress indicators like cortisol and glucose levels, and notable histopathological changes in gill and liver tissues. This research underscores the detrimental impact of plastic pollution on aquatic organisms, thereby highlighting potential risks to human health via the food chain (Zaman et al. 2024). A nutritionally balanced diet is essential for the health and growth of fish. The appropriate addition of micronutrients to fish feed can significantly contribute to achieving this nutritional balance. In this context, nanotechnology offers substantial advantages, as NPs exhibit enhanced bioavailability and can serve as effective micronutrient sources in fish diets (Singh, Panigrahi et al. 2024). This advancement has the potential to revolutionize aquaculture and ornamental fish farming practices. However, research on the optimal concentrations of micronutrients and NPs used as micronutrient sources in fish diets remains limited. Additionally, zebrafish that consume ZnONPs alongside their basal diet demonstrate superior growth and development, as ZnONPs facilitate significant feed intake and absorption, while also augmenting haematological components (Singh, Panigrahi et al. 2024). The combined supplementation of ZnONPs and SeNPs in equal ratios enhanced the expression of growth‐related genes and reduced the formation of ROS within zebrafish cells (Singh, Panigrahi et al. 2024). Thus, the synergistic use of ZnONPs and Se NPs as dietary supplements effectively promotes growth and supports the healthy development of zebrafish (Singh, Panigrahi et al. 2024). A 9‐week feeding trial investigated the effects of Fe‐NPs and CuNPs on the growth and health of channel catfish (Silva et al. 2024). The study compared diets supplemented with Fe‐NPs alone, CuNPs alone, a combination of both, and a control diet containing inorganic iron and copper sources (Silva et al. 2024). The evaluation of growth performance, haematological parameters, body composition and intestinal microbiota revealed no significant differences in production performance or survival rates after a bacterial challenge (Silva et al. 2024). However, fish receiving CuNPs exhibited significantly lower haematocrit and red blood cell counts compared to the control group, as well as an increased abundance of gram‐positive bacteria in their gut. Although Fe‐NPs did not influence haematological parameters, the results emphasized the essential role of dietary iron in haematopoiesis for channel catfish (Silva et al. 2024). Aquaculture represents a rapidly growing field within agriculture, significantly contributing to the global supply of animal protein while fostering beneficial interactions with the surrounding ecosystem (Moss et al. 2018). The success of fish farming is largely determined by two fundamental elements: the quality of feed and the conditions of the aquatic environment (Moller et al. 2023). Ensuring an optimal formulation of feed is essential for proper nutrition, whereas maintaining high water quality is vital to create a supportive habitat for fish development and survival (Hossain et al. 2022; Jewel et al. 2023; Akter et al. 2024). Recent studies indicate that NPs can significantly enhance growth and immune responses in fish (Kumar et al. 2023; Kumar, Thorat, Gunaware et al. 2024). ZnNPs are particularly highlighted for their positive effects on fish health and growth promotion (Jewel et al. 2024). These NPs provide numerous advantages, including environment‐friendly characteristics and a range of applications, such as in anticancer treatments and antimicrobial functions (Jiang et al. 2018). Consequently, a study was conducted to investigate the impact of ZnNPs supplementation on the growth and physiological performance of the catfish species, *Clarias batrachus* (Jewel et al. 2024). Findings indicated that fish receiving a diet enriched with 40 mg/kg of ZnNPs exhibited statistically significant enhancements in growth metrics, nutritional indices and haematological parameters compared to control group (Jewel et al. 2024). The optimal ZnNPs dietary supplementation for maximizing BWG and growth rate was determined to be between 30.4 and 30.5 mg/kg (Jewel et al. 2024). Furthermore, in a separate investigation, the effectiveness of green‐synthesized SeNPs was assessed, utilizing the microalga *Pediastrum boryanum* as a dietary supplement in aquaculture (Al‐Wakeel et al. 2024). This approach aimed to enhance the growth performance, overall health and immune response of Nile tilapia. In this study, the effects of varying concentrations of green‐synthesized SeNPs (79.26 nm) on Nile tilapia were investigated, with dosages of 0, 0.75 and 1.5 mg/kg administered over an 8‐week period (Al‐Wakeel et al. 2024). Results showed a significant enhancement in growth performance and innate immune parameters, including IgM and lysozyme levels, at both dosages compared to the control group (Al‐Wakeel et al. 2024). Notably, the supplementation did not induce any inflammatory response, as evidenced by stable pro‐inflammatory cytokine gene expression and histological examination of liver and spleen tissues, which showed no abnormalities (Al‐Wakeel et al. 2024). Overall, their findings suggest that green SeNPs can improve growth and immune responses in Nile tilapia, highlighting their potential as eco‐friendly dietary supplements in aquaculture (Al‐Wakeel et al. 2024). In aquaculture, fish encounter various stressors, including climate fluctuations and infectious diseases that hamper their performance, immune functions and overall welfare. Freshwater fish subjected to salt baths experience fatigue and heightened stress levels. Nile tilapia (*Oreochromis niloticus*) stands out as a widely cultivated species globally and has become one of the most sought‐after aquatic organisms. Salt treatment in freshwater fish is commonly utilized to mitigate a range of parasitic pathogens, from protozoans to helminths, due to its advantages of lower toxicity and cost‐effectiveness compared to more frequently employed anti‐parasitic agents like formalin and malachite green, thereby establishing it as the preferred therapeutic approach (Lio‐Po and Lim 2002; Schelkle et al. 2009). However, certain adverse effects of salt treatment have been noted, including negative impacts on osmoregulation, dehydration and potential immunosuppression, all of which are influenced by the treatment's duration and concentration (Tort 2011; Schmitz et al. 2017). Chitosan, a natural polymer derived from crustacean exoskeletons, is gaining attention due to its immune‐stimulant properties, biodegradability and compatibility, making it an excellent candidate for functional fish feeds and as a coating for medicinal substances and vaccine encapsulation (Zaki et al. 2015). CNPs exhibit enhanced bioavailability in the bloodstream, facilitating greater absorption at lower concentrations (Li et al. 2018). Vitamin C, an essential micronutrient for fish health, cannot be synthesized by fish and thus must be included in their diets. Nevertheless, it is sensitive to high temperatures, oxygen and light (Luis et al. 2021). Its antioxidant attributes help protect cellular structures from ROS, thereby promoting overall health, immune stimulation, anti‐aging and antimicrobial effects (Sherif and Zommara 2023). VE is another critical antioxidant that aids in safeguarding fish cells and tissues from ROS‐related damage during stressful conditions (Udo and Afia 2013). Research has demonstrated the immune‐boosting properties of VE, an essential fat‐soluble micronutrient for fish. Specifically, alpha‐tocopherol, one of the eight forms of VE, is commonly incorporated into fish feed due to its significant health benefits. It is noted to enhance both innate and adaptive immunity, reduce mortality rates and improve growth performance, while also maintaining the functional integrity of fish leukocytes (Sahoo and Mukherjee 2002). Therefore, a study was conducted to evaluate the effects of chitosan‐based NPs containing vitamins C and E (CCE‐NPs) on the physiological and immune responses of Nile tilapia subjected to a salt bath treatment of 30 ppt for 30 min daily (Elnagar et al. 2024). Fish were divided into groups based on whether they received CCE‐NPs for 7 or 14 days before or after the salt exposure. Results indicated that control fish showed significant increases in cortisol (5.44 µg/dL) and glucose levels (91.67 mg/dL) 1 h and 24 h post‐treatment. In contrast, tilapia consuming CCE‐NPs exhibited reduced cortisol and glucose levels, demonstrating better recovery of normal feeding behaviour by 72 h post‐treatment (Elnagar et al. 2024). Moreover, CCE‐NPs enhanced the expression of antioxidant genes and improved mucus lysozyme levels, contrasting with the control group, which did not return to baseline until 7 days later. Additionally, lower mortality rates and enhanced resistance to *S. agalactiae* infections were observed in tilapia fed CCE‐NPs (Elnagar et al. 2024). The findings suggest that dietary supplementation with CCE‐NPs may effectively bolster the immune response and mitigate stress‐induced susceptibility in Nile tilapia (Elnagar et al. 2024). The current phenomenon of global warming presents a major risk to ecosystems around the globe. This alteration in climate has also influenced the levels of pollution in marine environments, which in turn has consequences for human health (Kumar, Thorat, Chavhan et al. 2024). To address these issues, a study was conducted to evaluate the effects of Fe‐NPs on the toxicity of arsenic and ammonia, as well as high‐temperature stress in fish. Fe‐NPs were synthesized from fish waste and added to feed at concentrations of 10, 15 and 20 mg/kg. The experiment employed a completely randomized design with 12 treatments involving 540 fish. Notable findings included a significant reduction in cortisol levels at the 15 mg/kg dosage, alongside the modulation of various gene expressions related to stress response, immunity and growth. For instance, stress‐induced genes were downregulated, whereas immunity‐related genes showed increased expression, indicating improved immune responses. Additionally, the use of Fe‐NPs not only enhanced arsenic detoxification but also reduced mortality from bacterial infections, thereby underscoring the potential of dietary Fe‐NPs in promoting fish health under stress (Kumar, Thorat, Gunaware et al. 2024). Se is essential for several physiological functions in fish, including promoting optimal growth, development and providing antioxidant protection. Factors such as the specific form of Se, the composition of the diet, the cultivation environment, species of fish, developmental stage, health status and body size can significantly influence the effectiveness of dietary Se (Dawood et al. 2021; Khalil et al. 2022; Eissa et al. 2023). Recently, interest in the use of nano trace elements in aquaculture feed has surged due to their enhanced bioavailability (Dawood et al. 2021; Khalil et al. 2022). SeNPs are characterized by improved chemical stability, reduced toxicity and a capacity to gradually release Se post‐ingestion, which facilitates the more efficient synthesis of selenoproteins within the organism (Dawood et al. 2021; Khalil et al. 2022; Mohtashemipour et al. 2024). Furthermore, in another study, selenium‐CNPs (SeChNPs) were chemically synthesized and incorporated into specially formulated fish (Nile tilapia) feed, which contained 31.49 g of carbohydrates and 41.52 g of proteins (Srinivasan et al. 2024). Dietary supplementation with SeChNPs significantly enhanced the specific growth rate of Nile tilapia (*O. niloticus*) fingerlings, as indicated by greater BWG and better feed efficiency. FTIR spectroscopy confirmed the presence of Se and chitosan in the SeChNPs, identified by the functional groups at 3226, 2878 and 1734 cm^−1^ (Srinivasan et al. 2024). Additionally, scanning electron microscopy and energy dispersive x‐ray spectroscopy analysis identified a fibrous structure composed mainly of carbon, oxygen and Se (Srinivasan et al. 2024). The enhanced protein content of the SeChNP‐infused fish feed underscores its potential as a valuable growth promoter for fish. This innovative approach warrants further development and testing to optimize its efficiency as an effective fish feed supplement (Srinivasan et al. 2024). In another study, the effects of SeNPs on growth performance, survival rates, chemical composition and Se bioaccumulation in the muscle of Nile tilapia (*O. niloticus*) were assessed (Sheikh et al. 2023). After 56 days of feeding fish with average weights of 33.1 ± 1.0 g across four dietary treatments (control, T1, T2 and T3 corresponding to 0, 0.5, 1 and 2 mg/kg SeNPs), it was found that the diet containing 2 mg/kg SeNPs significantly enhanced BW increase, specific growth rate, and FCR compared to the control and T1 groups (Sheikh et al. 2023). Furthermore, survival rates improved notably in the T2 group relative to the control. The fish fed the higher SeNPs concentrations also exhibited statistically significant increases in protein, ash and moisture content in their muscle compared to the control group (Sheikh et al. 2023). The highest bioaccumulation of Se in muscle tissue was observed in the group receiving 2 mg/kg of SeNPs. Overall, SeNPs considerably enhanced the growth metrics, survival and nutritional quality of Nile tilapia, particularly in the T2 and T3 dietary treatments (Sheikh et al. 2023). Additionally, in another study, the effects of nutritional SeNPs on the growth and physiological and immunological parameters, as well as the antioxidant defence and stress response in fingerling Arabian yellowfin seabream (*Acanthopagrus arabicus*), were investigated (Abdollahi‐Mousavi et al. 2024). Fish weighing an average of 5 g were allocated evenly into 12 tanks, representing four experimental groups receiving diets with varying SeNP concentrations: 0 (control), 0.5, 1 and 2 mg/kg for 60 days. Results indicated that diets containing 1 and 2 mg/kg SeNPs enhanced growth metrics and feed efficiency (Abdollahi‐Mousavi et al. 2024). Notably, the 1 mg/kg SeNPs diet boosted the expression of genes related to growth and immunity. Although there were no significant differences in SOD activity and MDA levels, catalase activity increased in the 1 mg/kg group, along with heightened GSH peroxidase activity in the 0.5 and 1 mg/kg groups (Abdollahi‐Mousavi et al. 2024). Additionally, the 1 mg/kg group exhibited improved immune parameters and favourable lipid profiles, with increased high‐density lipoprotein and decreased low‐density lipoprotein and cholesterol levels. Both 0.5 and 1 mg/kg diets lowered plasma AST levels, whereas the 2 mg/kg diet elevated them. Furthermore, SeNPs effectively reduced blood cortisol and lactate concentrations in fish exposed to acute stress (Abdollahi‐Mousavi et al. 2024). Overall, the findings support the use of 1 mg/kg SeNPs to promote growth, immunity and stress resilience in Arabian yellowfin seabream (Abdollahi‐Mousavi et al. 2024). Other research demonstrated that the growth parameters, nutrient assimilation and mineral profile of *Catla catla* were markedly enhanced when fed diets based on soybean meal supplemented with SeNPs, with an optimal supplementation level determined to be 1.5 mg/kg (Ahmad et al. 2024). Consequently, further findings indicate that the supplementation of green‐synthesized SeNPs can significantly enhance the immune response of Nile tilapia and support their intestinal health (Zahran et al. 2024). In another study, it was found that dietary supplementation of manganese NPs (Mn‐NPs) at a concentration of 2 mg/kg alleviates lead and ammonia stress in fish while enhancing their growth performance under multiple stress conditions (Nalage et al. 2024). Additionally, this level of Mn‐NPs downregulated the gene expression of heat shock protein 70 (HSP 70) and cytochrome P450 (CYP 450) in response to stress, and it significantly reduced the bioaccumulation of lead in the tissues of fish exposed to lead and ammonia toxicity (Nalage et al. 2024). Furthermore, Mn‐NPs at 2 mg/kg effectively regulate apoptosis and the expression of the inducible nitric oxide synthase gene in fish subjected to various stressors (Nalage et al. 2024). The results of another study indicated that silica NPs (SiNPs) enhanced growth performance, feed utilization and final product quality in experimental fish (Bashar et al. 2021). In another study aimed at investigating the effects of varying amounts of SeNPs as a natural antioxidant and metabolic regulator on growth performance, antioxidant capacity, digestive enzymes and immune resistance in *O. niloticus* challenged with *A. flavus* infection, it was concluded that SeNPs at levels up to 1.0 mg/kg diet can be effectively used in tilapia feed to enhance fish production, immune system response and histopathological parameters (Eissa et al. 2023). Heavy metals, particularly Cd, are among the most significant and persistent pollutants found in wastewater, characterized by their non‐biodegradable nature pollution (Abdel‐Tawwab et al. 2024). Cd enters aquatic ecosystems primarily through the discharge of industrial and agricultural waste, resulting in considerable harm to the health and welfare of aquatic organisms’ pollution (Abdel‐Tawwab et al. 2024). Additionally, the application of feed supplements enriched with immune‐stimulants presents a compelling approach to alleviate the toxic effects of heavy metals, including Cd. Hence, in a study aimed at assessing the effects of bio‐synthesized CNPs derived from Bacillus subtilis (Bs‐CNPs) on Cd‐intoxicated Nile tilapia (O. niloticus), various treatment combinations of Bs‐CNPs and Cd exposure were evaluated over 60 days (Abdel‐Tawwab et al. 2024). The findings demonstrate that Cd exposure significantly hindered growth, digestive enzyme function and survival rates of the fish, with the most severe effects observed at higher Cd concentrations. Conversely, diets supplemented with Bs‐CNPs notably improved growth performance and physiological parameters in tilapia, even in the presence of Cd, indicating a protective effect against heavy metal toxicity pollution (Abdel‐Tawwab et al. 2024). These results suggest that administering Bs‐CNPs could be a viable strategy for enhancing the health and well‐being of Nile tilapia in environments affected by Cd pollution (Abdel‐Tawwab et al. 2024). The fortification of fish with synthesized ZnONPs shows promise as a food additive for reducing microbial spoilage and lipid peroxidation during storage (Mahato et al. 2024). The supplementation of curcumin NPs enhanced growth, feed intake, digestion, haematological and biochemical indicators, immune response and redox balance in *Dicentrarchus labrax* (Bin‐Ammar et al. 2024). Thus, curcumin NPs can be considered a natural option in the diets of *D. labrax* to promote overall performance (Bin‐Ammar et al. 2024).

Furthermore, supplementing the diet with SeNPs at concentrations of 1–5 ppm led to an increase in antioxidant capacity and enhanced lysozyme activity in the larvae of the Arabian yellowfin seabream (*A. arabicus*) (Jafari et al. 2024). Additionally, it reduced lipid peroxidation throughout their bodies. Furthermore, higher levels of SeNPs in the diet corresponded to an increase in docosahexaenoic acid (Jafari et al. 2024). The results of the study indicate that the implementation of CNPs loaded with *Mentha piperita* essential oil may promote the growth and strengthen the immune responses of Siberian sturgeon fry, thereby facilitating a more sustainable approach to production within intensive aquaculture systems (Adel et al. 2024). In a different investigation, the application of organic Zn, whether in NPs or bulk form, was found to enhance the population of immune cells, specifically lymphocytes and neutrophils, in response to bacterial infections (Silva et al. 2024). Nevertheless, other assessed parameters in Nile tilapia did not show any significant variation depending on the size or form of the Zn (organic vs. inorganic) (Silva et al. 2024). In the aquaculture sector, SiNPs play a crucial role, particularly in disease management. When compared to other advanced nanomaterials, SiNPs exhibit superior stability in terms of chemical, mechanical and thermal properties, withstanding temperatures of up to 1500°C, and they possess a higher concentration of hydroxyl groups (Singh, Panigrahi et al. 2024). In this context, recent studies suggest that SiNPs represent an innovative and effective method for mitigating immunological, antioxidant, physiological and histopathological changes caused by infection with *Aeromonas veronii* (Mahboub et al. 2023). Exposure to NPs at concentrations exceeding normal levels can induce toxicity in aquatic organisms. A zebrafish embryo study revealed that even biologically synthesized ZnONPs (from *Sargassum polycystum*; 100 nm rod‐shaped particles) caused significant toxic effects, including developmental impairments (reduced heart rate, hatching delay), dose‐dependent teratogenicity, oxidative stress (oxidant/antioxidant imbalance), disruption of Na+/K+‐ATPase and acetylcholinesterase activities and increased apoptosis (Deenathayalan et al. 2024). This demonstrates that green synthesis does not eliminate NPs toxicity risks.

## 8 Conclusion

The integration of nanotechnology into veterinary science represents a significant advancement in enhancing animal health and nutrition. The unique properties of NPs offer innovative solutions for improving nutrient delivery, disease prevention and mycotoxin reduction, ultimately leading to better livestock productivity and welfare. Evidence suggests that NP‐encapsulated nutrients significantly enhance absorption rates compared to conventional formulations, which can lead to improved gut health and overall animal performance. Moreover, NPs have been shown to stabilize and enhance the effectiveness of probiotics, contributing to better immune responses in various animal species. The application of NPs in vaccines has also demonstrated improved immunogenicity, which is crucial for effective disease control in livestock. However, it is crucial to address safety and toxicity concerns to ensure responsible use in animal applications. The potential risks associated with NPs must be thoroughly evaluated to safeguard animal health and ensure consumer safety. Continued research and collaboration among scientists, veterinarians and industry stakeholders will be essential in translating these findings into practical solutions for the agricultural sector. By harnessing the benefits of nanotechnology while mitigating risks, the veterinary field can pave the way for more sustainable and efficient livestock production systems in the future.

## Author Contributions

Mohsen Kazemi is the sole author of this review article. He conducted a comprehensive literature review, synthesized information from various sources and critically analysed existing studies. All aspects of the article have been thoroughly reviewed and approved by the author.

## Conflicts of Interest

The author declares no conflicts of interest.

## Data Availability

The data used in this review article are derived from reputable sources and do not include any original data from the author.
